# Mitochondrial Genome Sequences and Structures Aid in the Resolution of *Piroplasmida* phylogeny

**DOI:** 10.1371/journal.pone.0165702

**Published:** 2016-11-10

**Authors:** Megan E. Schreeg, Henry S. Marr, Jaime L. Tarigo, Leah A. Cohn, David M. Bird, Elizabeth H. Scholl, Michael G. Levy, Brian M. Wiegmann, Adam J. Birkenheuer

**Affiliations:** 1 North Carolina State University, College of Veterinary Medicine, Raleigh, North Carolina, United States of America; 2 University of Georgia, College of Veterinary Medicine, Athens, Georgia, United States of America; 3 University of Missouri, College of Veterinary Medicine, Columbia, Missouri, United States of America; 4 North Carolina State University, College of Agriculture and Life Sciences, Raleigh, North Carolina, United States of America; University of the Sunshine Coast, AUSTRALIA

## Abstract

The taxonomy of the order *Piroplasmida*, which includes a number of clinically and economically relevant organisms, is a hotly debated topic amongst parasitologists. Three genera (*Babesia*, *Theileria*, and *Cytauxzoon*) are recognized based on parasite life cycle characteristics, but molecular phylogenetic analyses of 18S sequences have suggested the presence of five or more distinct *Piroplasmida* lineages. Despite these important advancements, a few studies have been unable to define the taxonomic relationships of some organisms (e.g. *C*. *felis and T*. *equi*) with respect to other *Piroplasmida*. Additional evidence from mitochondrial genome sequences and synteny should aid in the inference of *Piroplasmida* phylogeny and resolution of taxonomic uncertainties. In this study, we have amplified, sequenced, and annotated seven previously uncharacterized mitochondrial genomes (*Babesia canis*, *Babesia vogeli*, *Babesia rossi*, *Babesia* sp. Coco, *Babesia conradae*, *Babesia microti*-like sp., and *Cytauxzoon felis*) and identified additional ribosomal fragments in ten previously characterized mitochondrial genomes. Phylogenetic analysis of concatenated mitochondrial and 18S sequences as well as *cox1* amino acid sequence identified five distinct *Piroplasmida* groups, each of which possesses a unique mitochondrial genome structure. Specifically, our results confirm the existence of four previously identified clades (*B*. *microti* group, *Babesia* sensu stricto, *Theileria equi*, and a *Babesia* sensu latu group that includes *B*. *conradae*) while supporting the integration of *Theileria* and *Cytauxzoon* species into a single fifth taxon. Although known biological characteristics of *Piroplasmida* corroborate the proposed phylogeny, more investigation into parasite life cycles is warranted to further understand the evolution of the *Piroplasmida*. Our results provide an evolutionary framework for comparative biology of these important animal and human pathogens and help focus renewed efforts toward understanding the phylogenetic relationships within the group.

## Introduction

Parasites in the order *Piroplasmida*, which include *Babesia*, *Theileria*, and *Cytauxzoon* species, cause important diseases across the globe in humans, livestock, wildlife, and companion animals [1–7]. Despite the clinical and economic importance of these tick-transmitted parasites, the taxonomic relationships between many *Piroplasmida* remain ambiguous, which is problematic when attempting to understand and treat the diseases they cause. Classical taxonomy of *Piroplasmida* has been based on mechanisms of transmission in the tick host, host cell type(s) infected, and sometimes parasite morphology and vertebrate host preference [7–13]. *Theileria* and *Cytauxzoon* species are limited to transstadial transmission in the tick and initially infect nucleated cells within the vertebrate host [7,13,14]. Alternatively, *Babesia* species have acquired character traits that presumably enhance their propagation, including transovarial transmission in the tick and exclusive infection of erythrocytes in the vertebrate host [15]. However, as more molecular and biological information has been discovered, it has become apparent that this classification scheme is limited and fails to reflect the diversity and evolution of the *Piroplasmida*.

**Fig 1:**
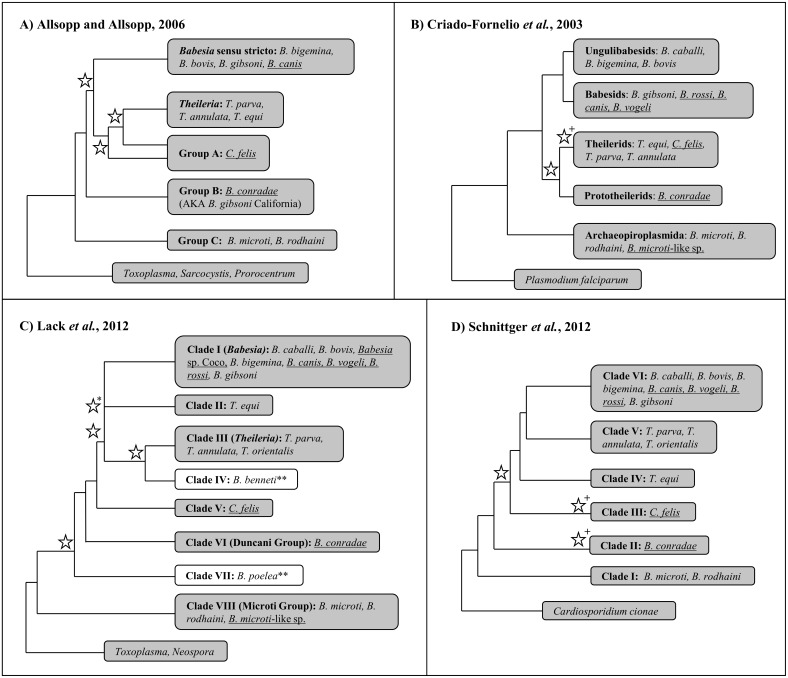
18S sequence alone is unable to completely resolve phylogeny of the *Piroplasmida*. Summary topologies of *Piroplasmida* phylogenetic trees based on 18S rRNA sequence analyses in four previously reported studies [7, 9–11]. The nomenclature of sub-groupings assigned in each study is maintained in each individual tree. Stars denote nodes that had less than 70% bootstrap support (A-C) or less than 95% Bayesian posterior probability (C-D); cut-off values for bootstrap values and posterior probabilities reflect those utilized by Lack *et al*. Individual species included in each respective study whose mitochondrial genomes were utilized in this study are noted within each clade. Mitochondrial genomes first characterized in this study are underlined. +Low support for species included within clade. *Although Lack *et al*. found strong support for a node uniting Clade I-III, positioning of Clade II with respect to Clades I and III within this node was unresolved. **White clades in 1C indicate those for which no representative species were characterized in this study; Clade IV includes *Babesia benneti*, while Clade VII include *Babesia poelea*.

Historically, one of the most important and widely used criterion for categorizing *Babesia* species has been describing the parasite morphology as either “large” or “small” based on relative piroplasm size. However, as more molecular data (DNA sequences) have been acquired for *Babesia* species, it has become clear that similar piroplasm morphology does not equate to genetic relatedness [7,9–11,15,16]. Given this discrepancy between molecular data and parasite morphology, extensive efforts have been made to further analyze molecular data for a number of *Babesia* species. The results of these analyses resoundingly indicate that the currently recognized genus *Babesia* is paraphyletic [17–20]. Consequently, *Babesia* species have been informally divided into *Babesia* sensu stricto (s.s.) and *Babesia* sensu latu (s.l. [7]). *Babesia* s.s. refers to *Babesia* as classically defined, and likely represents a monophyletic group of organisms that are transovarially transmitted in tick hosts and only infect erythrocytes. *Babesia* s.l., however, refers to species that morphologically resemble *Babesia* but either have schizogony in the vertebrate host, lack transovarial transmission in the tick host, or cannot equivocally be assigned to either *Babesia* or *Theileria* [7,9,11,18,19,21–23]. *Babesia* s.l. likely includes at least two subgroups: the “Archaeopiroplasmida/Microti” group, which includes the extensive *Babesia microti* complex [10,11,21], and the “Prototheilerids/Duncani/Western” group, which primarily includes multiple organisms identified in the Western United States [10,11,18,22,23]. Phylogenetic analyses of a variety of targets (18S, ITS, Beta tubulin) indicate that the *Babesia microti* complex represents a distinct lineage (Fig 1; [7,9–11,21,24]). Additionally, most molecular phylogenetic analyses suggest that members of *Babesia* s.l. originated earlier than *Babesia* s.s. and *Theileria* [7,9–11,24]. However, with the exception of *B*. *microti* [25], putatively “primitive” morphological features (invasion of nucleated cells) have not been detected for these organisms [22,23].

Similarly, discrepancies between molecular and morphological data have complicated the classification of *Theileria* and *Cytauxzoon* species. *Theileria* sensu stricto consists of a monophyletic clade of organisms possessing traditional *Theileria* biological features. A number of species that do not fall into the *Theileria* sensu stricto clade would be more properly designated as *Theileria* sensu latu, including *T*. *equi*, *T*. *youngi*, and *T*. *bicornis*. *Cytauxzoon* was originally established as a distinct genus from *Theileria* due to its invasion of monocytes rather than lymphocytes [14]. However, species in both genera were later shown to infect both lymphocytes and monocytes [26–28], and consequently the majority of *Cytauxzoon* species were reclassified as *Theileria* [26,29–34]. Despite the suggestion to completely eliminate the genus *Cytauxzoon* [33], *Cytauxzoon felis* was never reclassified as *Theileria*, and for decades was the lone species recognized within the genus. Unfortunately, molecular data has only confused this situation further, as a number of novel parasites have been classified as either *Theileria* or *Cytauxzoon* on the basis of molecular data (18S rRNA sequence) alone, despite there being no evidence of invasion of nucleated cells for any of these species [35–40]. It should be noted that an exhaustive search for schizogony was not performed in any of these studies [35–40]. Furthermore, phylogenetic analyses of 18S have failed to agree on a definitive taxonomic placement for *Cytauxzoon* species within *Piroplasmida* (Fig 1; [7,9–11,24]).

Since its discovery, the taxonomy of *Theileria equi* has also been strongly debated [7–11,24,30,41–43]. Originally named *Babesia equi*, *T*. *equi* was reclassified within *Theileria* upon discovery of lymphocyte and monocyte invasion [41,44]. However, initial molecular phylogenetic analyses of 18S were unable to confirm the placement of *T*. *equi* within *Theileria* [8–10], and in fact, the majority of recent molecular phylogenetic analyses of 18S demonstrate that *T*. *equi* represents a unique clade within *Piroplasmida* (Fig 1; [7,11,24,45,46]). Furthermore, two of these studies have placed *T*. *equi* as a sister group to the clade comprised of *Babesia* s.s. and *Theileria* s.s. [7,45]. This notion has been further corroborated by a phylogenetic analysis of 150 putative protein sequences [42]. Together, these studies indicate that *T*. *equi* would most appropriately be recognized as a genus separate from *Theileria* and *Babesia*.

There is a need for additional approaches in conducting molecular phylogenetic analyses of *Piroplasmida*. The majority of previous analyses have used 18S rRNA sequence alone to estimate phylogenetic relationships. However, the complexity of 18S secondary structure has led to inconsistencies in gene alignment across studies [47]. Additionally, alignment algorithms, evolutionary models, optimality criterion, statistical analysis methods, and number/types of species included in datasets varies widely between different studies (Fig 1; [7,9–11,24,30]). As a result, phylogenetic analyses of *Piroplasmida* do not agree on the taxonomic placement of some species, and some studies have had low statistical support for some recovered clades (Fig 1; [7,9–11,24,30]). As an alternative to analyzing 18S rRNA sequences alone, mitochondrial genome sequences and structures have proven to be useful for elucidation of evolutionary relationships and for delineating specimens to the species level for a number of eukaryotes, including closely related protozoan parasites [48–62]. Mitochondrial genomes should provide important new evidence for resolving *Piroplasmida* phylogeny.

Here, we describe the sequence and gene order of seven previously uncharacterized *Piroplasmida* mitochondrial genomes, including annotation of protein-encoding genes cytochrome *b* (*cytb*) and cytochrome *c* oxidase subunits I and III (*cox1* and *cox3*) as well as ribosomal subunit fragments. Additionally, we have identified conserved ribosomal subunit fragment sequences from 10 previously reported *Piroplasmida* mitochondrial genomes. Phylogenetic analysis of these mitochondrial genome sequences concatenated along with 18S sequences identify five distinct *Piroplasmida* lineages with strong statistical support: 1) *Babesia* sensu stricto, 2) *Theileria* and *Cytauxzoon*, 3) *Theileria equi*, 4) Western *Babesia* group (represented by *B*. *conradae*), and 5) the *Babesia microti* group. These five groups, which can also be identified by analysis of *cox1* amino acid sequence, are further supported by unique mitochondrial genome structural arrangements as well as previously reported and newly characterized biological features.

## Materials and Methods

### Parasite species

Mitochondrial genomes were characterized for seven *Piroplasmida* species that commonly infect companion animals (dogs and cats; Table 1). Blood samples previously confirmed to be infected with these parasites (18S amplification and sequencing) were obtained from the North Carolina State University College of Veterinary Medicine Vector-Borne Disease Diagnostic Laboratory. One infected blood sample was utilized for each mitochondrial genome characterized. All additional parasite sequences utilized in this study are summarized in Table 1.

**Table 1:** Species and sequences utilized in phylogenetic analysis.

Species	Current Categorization^a^	Host Effected	MT Genome GenBank Accession Number	18S GenBank Accession Number
*Babesia caballi*	*Babesia sensu stricto*	Equine	AB499086	Z15104
*Babesia bigemina*	*Babesia sensu stricto*	Bovine	AB499085	HQ840960
*Babesia bovis*	*Babesia sensu stricto*	Bovine	AB499088	AY150059
*Babesia canis*	*Babesia sensu stricto*	Canine	KC207822^c^	AY072926
*Babesia rossi*	*Babesia sensu stricto*	Canine	KC207823^c^	L19079
*Babesia vogeli*	*Babesia sensu stricto*	Canine	KC207825^c^	AY072925
*Babesia conradae*	*Babesia sensu latu* (Western *Babesia* group?)	Canine	KC207826^c^	AF158702
*Babesia gibsoni*	*Babesia sensu stricto*	Canine	AB499087	EU583386
*Babesia microti*	*Babesia sensu latu* (*Babesia microti* group?)	Murine, Human	FO082868, AB624353^d^	U09844
*Babesia microti*-like sp. (syn. *Babesia vulpes*, *Theileria annae*, *Babesia* cf. *microti*) ^b^	*Babesia sensu latu* (*Babesia microti* group?)	Canine	KC207827^c^	AF188001
*Babesia rodhaini*	*Babesia sensu latu* (*Babesia microti* group?)	Murine	AB624357	M87656
*Babesia sp*. Coco	*Babesia sensu stricto*	Canine	KC207824^c^	EU109716
*Cytauxzoon felis*	Unclear: *Theileria*? Unique group?	Feline	KC207821^c^	AY679105
*Theileria annulata*	*Theileria* sensu stricto	Bovine	NW001091933	M64243
*Theileria equi*	Unclear: *Theileria*? Unique group?	Equine	AB499091	EU642511
*Theileria orientalis*	*Theileria* sensu stricto	Bovine	AB499090	HM538266
*Theileria parva*	*Theileria* sensu stricto	Bovine	AB499089	L02366
*Plasmodium falciparum*	N/A	Human	AY283019	Z23263

^a^Names of sub-groups as previously defined [7, 11]

^b^*Babesia microti*-like sp. isolated from foxes and Spanish dogs is also referred to in the literature by a number of alternative names. This organism will be referred to as *Babesia microti*-like sp. in this manuscript due to a recent publication classifying alternative names as unavailable [63].

^c^Denotes mitochondrial genomes that were first characterized in this study

^d^Two mitochondrial genomes that vary in sequence and structure have been reported for *B*. *microti*; both are utilized in this study

### Samples and DNA isolation

DNA was extracted from 200 μL of anti-coagulated infected whole blood samples using a commercial kit according to manufacturer’s instructions (QIAamp DNA Blood Mini Kit, Qiagen Inc., Valencia, CA). All blood samples had originally been submitted for diagnostic purposes and would otherwise have been discarded.

### PCR amplification of mitochondrial genomes

Using conserved regions of previously reported *Piroplasmida* mitochondrial genomes as a guide [58], primers were designed to PCR-amplify near-full length mitochondrial genomes of *C*. *felis*, *B*. *canis*, *B*. *rossi*, *B*. *vogeli*, *B*. *conradae*, and *Babesia* sp. Coco in three overlapping fragments (Table 2, see S1 Fig). Additional PCR assays were designed as needed for each species to obtain additional mitochondrial genome sequence (see S1–S6 Tables and S1–S3 Figs). Each 50 μL reaction contained 1 μL of DNA template, 50 pmol of each primer, 10 nmol dNTPs, 75 nmol of MgCl_2_, 3.75 U AmpliTaq Gold DNA polymerase and a 1X concentration of GeneAmp PCR Gold Buffer (Applied Biosystems, Carlsbad, CA). Thermal cycling conditions consisted of an initial denaturation at 94°C for 5 minutes, followed by 40–50 amplification cycles (94°C for 20 seconds, 53–61°C for 30 seconds, and 68°C for 1–3.5 minutes) and a final extension step at 72°C for 7 minutes (Techne Inc., Burlington, NJ). Annealing temperatures were optimized as needed utilizing a temperature gradient, and extension times and cycle number varied with amplicon length.

**Table 2:** Primers utilized in PCR amplification of *Piroplasmida* mitochondrial genomes.

Amplicon	Forward Primer	Reverse Primer
**MT Genome Fragment 1**^a^	GGAAGTGGWACWGGWTGGAC	ACTTTGAACACACTGCTCG
**MT Genome Fragment 2**^a^	AGGCATGCAATACCGAACAGG	AAGGTACGCCRGGGATAACAGG
**MT Genome Fragment 3**^a^	AAGGTATGGTGAGACGACATGG	CTTAACCCAACTCACGTACC
***cox1***^b^^,^ ^c^	GGAAGTGGWACWGGWTGGAC	TTCGGTATTGCATGCCTTG
***cytb***^b^	TTAGTGAAGGAACTTGACAGGT	CGGTTAATCTTTCCTATTCCTTACG
***cox3***^b^	ACTGTCAGCTAAAACGTATC	ACAGGATTAGATACCCTGG
***cox3* (*Babesia microti* group)**^b^	CTCGATATTAATCTTAAAGTACAGGAC	ACTCATATCTATTACCACTATAGGC

^a^Primers were designed based on sequences of previously reported related *Piroplasmida* mitochondrial genomes. Three primer sets were utilized for the amplification of a near-full length mitochondrial genome for the majority of species (5 out of 7) characterized in this study. For additional primers used see S1–S7 Tables.

^b^After sequencing of the mitochondrial genomes was complete, primers were designed in highly conserved regions for amplification of partial *cox1* and full length *cytb* and *cox3* in all species, and are recommended for amplification of these genes in future studies^.^

^c^Recommended primer set for amplification of *cox1* for phylogenetic analysis

Sequences of the 5’ end of the *cox1* gene and mitochondrial telomeric regions were determined by inverted PCR [58]. Primer pairs directed at terminal inverted repeats (TIR) would presumably self-anneal, leading to amplification of the remainder of the mitochondrial genome (see S1 Fig and S1–S3 Tables). A proofreading DNA-polymerase with exonuclease activity (LA Taq) was used to remove any unpaired bases that would interfere with the inverted PCR self-annealing. Each 50 μL PCR reaction contained 1 μL of DNA template, 50 pmol of each primer, 10 nmol dNTPs, 2.5 U LA Taq DNA polymerase and a 10X concentration of LA PCR Buffer II plus Mg^+2^ (Takara Bio Inc., Shiga, Japan). Thermal cycling conditions consisted of an initial denaturation at 95°C for 5 minutes, followed by 40 amplification cycles (94°C for 20 seconds and 68°C for 2.25 minutes) and a final extension step at 72°C for 7 minutes. Amplicons produced from the inverted PCR reactions (*C*. *felis*, *B*. *canis*, *and B*. *rossi*) were directly cloned according to manufacturer’s instructions (pGEM-T Easy vector system, Promega, San Luis Obispo, CA) and transformed into TOP-10 competent *E*. *coli* (Invitrogen, Grand Island, NY). Plasmids containing inserts of the appropriate size were isolated according to manufacturer’s instructions (QIAprep Spin Miniprep Kit, Qiagen, Inc., Valencia, CA).

Due to the unique mitochondrial genome structure of *B*. *microti*-like sp., an alternative PCR approach was required to amplify a near-full length mitochondrial genome (see S4 Fig and S7 Table). Primers were designed based on previously reported mitochondrial genome sequences for *B*. *microti* (FO082868 and AB624353) and *B*. *rodhaini* (AB624357) [64,65]. The region of the mitochondrial genome containing *cox1* and *cytb* was amplified in four overlapping fragments, while the region of the mitochondrial genome containing *cox3* was amplified in two overlapping fragments. Each 50 μL reaction contained 1 μL of DNA template, 50 pmol of each primer, 10 nmol dNTPs, 75 nmol of MgCl_2_, 2.5 U AmpliTaq Gold DNA polymerase and a 1X concentration of GeneAmp PCR Gold Buffer (Applied Biosystems, Carlsbad, CA). Thermal cycling conditions consisted of an initial denaturation at 95°C for 5 minutes, followed by 45 amplification cycles (95°C for 20 seconds, 53–60°C for 30 seconds, and 68°C for 0.75–2 minutes) and a final extension step at 72°C for 7 minutes; amplification of Fragment 4 required modified extension temperature due to high AT content of the amplicon (60°C during cyclic extension and 68°C during final extension) [66]. Annealing temperatures were optimized as needed utilizing a temperature gradient, and extension times and cycle number varied with amplicon length. Because assumed internal inverted repeats [64,67] were not relevant for phylogenetic analyses, amplification and sequencing of these regions were not pursued.

Positive controls consisted of confirmed DNA extracted from *Babesia gibsoni*-infected canine whole blood and negative controls consisted of DNA extracted from uninfected canine whole blood and water (no DNA). For *B*. *microti*-like sp., *Babesia gibsoni*-infected canine whole blood was used when possible as a positive control (e.g. amplification of protein encoding genes); otherwise, for amplification of regions unique to *B*. *microti*-like sp., no appropriate positive control was available to our laboratory. All amplicons were confirmed via electrophoresis on ethidium bromide-stained 1% agarose (Genesee Scientific, San Diego, CA).

### Amplification of *B*. *conradae cox3*-like gene

Because a putative *cox3* gene was not found in the mitochondrial genome of *B*. *conradae*, primers were designed based on highly conserved sequence flanking the *cox3* gene in all *Babesia*, *Theileria*, and *Cytauxzoon* species referenced and examined in this paper (see S6 Table). A 700-bp amplicon was produced using PCR. Each 50 μL reaction contained 1 μL of DNA template, 50 pmol of each primer, 10 nmol dNTPs, 75 nmol of MgCl_2_, 2.5 U AmpliTaq Gold DNA polymerase and a 1X concentration of GeneAmp PCR Gold Buffer (Applied Biosystems, Carlsbad, CA). Thermal cycling conditions consisted of an initial denaturation at 94°C for 5 minutes, followed by 45 amplification cycles (94°C for 20 seconds, 50°C for 30 seconds, and 68°C for 1.5 minutes) and a final extension step at 72°C for 7 minutes. Positive and negative controls and assessment of amplicons were identical to other mitochondrial PCRs; no amplicons were produced from DNA extracted from uninfected canine blood.

### Sequencing

Purified amplicons (QIAquick PCR purification kit, Qiagen Inc., Valencia, CA) and plasmids were sequenced bi-directionally (MCLAB, South San Francisco, CA and Genewiz, South Plainfield, NJ). Additional primers were used for sequencing when necessary to obtain complete bi-directional sequence (see S1–S7 Tables). Sequence chromatograms were carefully inspected for heterogeneity, and contigs were assembled using the BioEdit Sequence Alignment Editor (North Carolina State University, Raleigh, NC). For those amplicons with sequence that could not be resolved directly (see S1–S7 Tables), PCR products were cloned using the pGEM-T Easy vector system as described above.

### Genome annotation

Protein-encoding genes (*cox1*, *cox3*, *cytb*) were identified by screening mitochondrial genomes for open reading frames. Putative genes were then queried against mitochondrial sequences of related parasites, and identified orthologs were aligned for confirmation. To identify putative rRNA gene fragments, mitochondrial sequences were queried against previously reported rRNA sequences from *T*. *parva* (Z23263 [68]) using blastn under default algorithm parameters (NCBI BLAST). For identification of rRNA fragments of *B*. *microti*-like sp., *B*. *rodhaini*, and *B*. *microti*, mitochondrial sequences for these species were further queried against rRNA sequences of *B*. *microti* (AB624353) kindly provided by Kenji Hikosaka [52]. Additionally, alignment of identified rRNA sequences with previously annotated mitochondrial genomes was utilized in determining termini of rRNA fragments as needed (ClustalW, BioEdit Sequence Alignment Editor, North Carolina State University, Raleigh, NC).

### Alignment, substitution model choice and phylogenetic inference

Alignments for mitochondrial and 18S sequences were carried out under standard configurations using ClustalW (BioEdit Sequence Alignment Editor, North Carolina State University, Raleigh, NC), and all sequences were “cropped” to the length of the shortest sequence in the alignment so as to prevent bias for nucleotides that were only amplified from select samples. Alignments were further edited, verified, and manually adjusted as needed in MEGA 6.05 [69]. Protein coding genes were translated to amino acids to guide nucleotide alignment and to construct amino acid data sets. Highly variable regions where positional homology was uncertain or *ad hoc*, especially in ribosomal RNAs, were identified by inspection and excluded from phylogenetic analyses. The resulting alignment for each gene was concatenated using SequenceMatrix v. 1.7.8 [70]; only those RNA fragments that were identified for all species were included (see S8 Table). Alignments and phylogenetic data sets are archived in the DRYAD public data repository (www.datadryad.org).

Nucleotide substitution models were chosen by PartitionFinder 1.1.1 [71] using the Bayesian Information Criterion (BIC). PartitionFinder was also used to find the best partitioning scheme based on BIC using the “greedy” option.

For phylogenetic tree reconstruction, two optimality criteria were used: Maximum Likelihood (ML) and Bayesian Analysis (MB). For the maximum likelihood analysis, we used RaxML v8 [72] on concatenated datasets. *Plasmodium falciparum* was set as the outgroup and 12345 was used as the random seed value. No secondary structure options were used. Tree inference was carried out under the GTRGAMMA model, and multiparametric bootstrapping was performed for 100–200 iterations. For Bayesian analysis, we used MrBayes 3.2.2 [73] with the MCMCMC (Metropolis coupled Markov Chain Monte Carlo) algorithm. According to results of likelihood ratio tests in PartitionFinder 1.1.1 [71], seven unique partitions were identified and each was independently estimated under a specific site model. We carried out two simultaneous runs using the standard configuration with eight chains for every 108 generations, saving a tree every 1000 generations after discarding a burnin of 25%. Complete sampling was analyzed using ML criterion without partitioning. Clade support was assessed by examining Bayesian posterior probabilities (PP) from a post-burnin sample of optimal trees. ML Bootstrap percentages were compared to Bayesian posterior probabilities for each node to inform clade support. Resulting tree topologies were visualized in Figtree v1.4 (http://tree.bio.ed.ac.uk/software/figtree/).

## Results

### Mitochondrial genome structures

Here, we present a detailed characterization of complete mitochondrial genome sequences from *B*. *rossi*, *B*. *canis*, and *C*. *felis*, and near-complete mitochondrial genome sequences from *B*. *vogeli*, *B*. *conradae* and *Babesia* sp. Coco, and *B*. *microti*-like sp.

**Fig 2:**
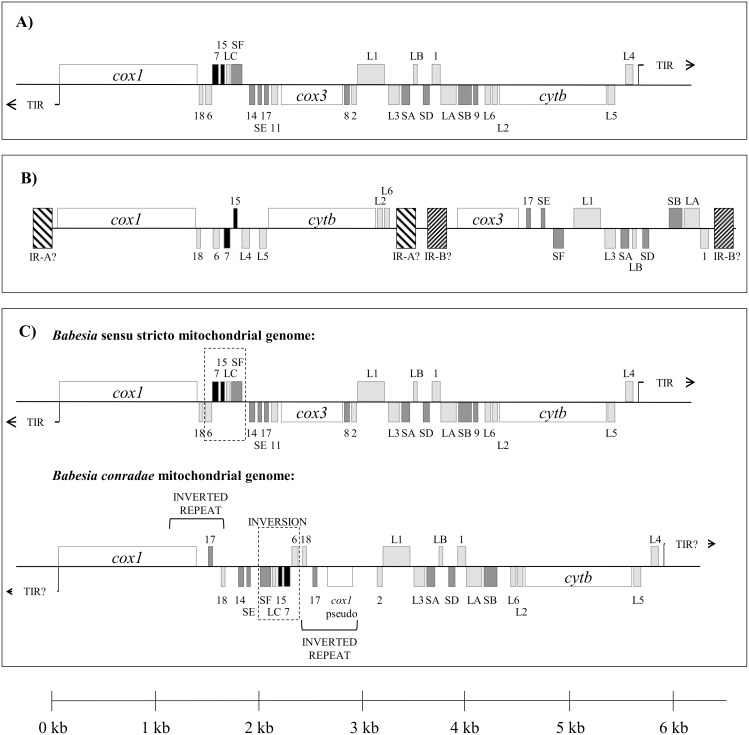
Mitochondrial genome structures of *Piroplasmida* species characterized in this study. Genes shown above the central line are coded on the sense strand, while those below are on the antisense strand. Protein-coding genes (*cox1*, *cox3*, and *cytb*) are indicated in white. Large subunit rRNA fragments are in light gray, small subunit rRNA fragments are in dark gray, and miscellaneous conserved RNA fragments are in black. A) Mitochondrial genome sequences of *C*. *felis*, *B*. *rossi*, *B*. *vogeli*, *B*. *canis*, and *Babesia* sp. Coco maintained the mitochondrial genome structure that is characteristic of traditional *Babesia sensu stricto* and *Theileria* species, while B) the inferred *Babesia microti*-like sp. mitochondrial genome structure appears to be similar to that of *B*. *microti* and *B*. *rodhaini*, suggesting it has a “flip-flop” mitochondrial genome structure. Assumed inverted repeats A and B (indicated as IR-A and IR-B) were not confirmed due to lack of relevant sequence for phylogenetic analysis. C) *Babesia conradae* had a unique mitochondrial genome, which lacked *cox3* and had a duplicated inversion that included the 3’ end of *cox1* and RNA17 and RNA18. Additionally, a collection of rRNA fragments (RNA6, RNA7, RNA15, LSUC, and SSUF) found in *Babesia* sensu stricto, *C*. *felis*, and *Theileria* mitochondrial genomes was conserved but inverted as a unit adjacent to the duplicated inversion.

*B*. *canis*, *B*. *rossi*, *B*. *vogeli*, *Babesia* sp. Coco, and *C*. *felis* shared mitochondrial genome organization with previously reported *Babesia* sensu stricto and *Theileria* species (Fig 2A) [58]. Linear mitochondrial genomes ranged in size from 5.6 to 5.9 kb, and included the protein-encoding genes *cox1*, *cox3*, and *cytb*, as well as multiple rRNA fragments (Fig 2A). Similar to related species, terminal inverted repeats (TIR) were identified and characterized for *B*. *rossi*, *B*. *canis*, and *C*. *felis* [58]. However, multiple attempts at inverted PCR were unsuccessful for *B*. *vogeli* and *Babesia* sp. Coco. Therefore, for these species the 5’ end of *cox1* could not be characterized and the presence or absence of TIRs could not be confirmed.

Two separate pieces of the *B*. *microti*-like sp. mitochondrial genome were amplified that collectively comprised 5.9 kb (Fig 2B). Organization of protein-coding genes and rRNA fragments on these fragments suggest that the *B*. *microti*-like sp. has a “flip-flop” mitochondrial genome structure similar to that of its close relative, *B*. *microti* [64,67]. Amplification spanning inverted repeats was not attempted due to lack of relevant sequence for phylogenetic analysis.

In contrast, *B*. *conradae* had a novel mitochondrial genome structure (Fig 2C). While it had similar size (5.6 kb) and organization of *cox1*, *cytb*, and some rRNA fragments as *Babesia* sensu stricto species, a *cox3* gene was not identified at the predicted location within the mitochondrial genome. Instead, between *cox1* and LSU1, there was an inverted duplication that included the 3’ end of *cox1* as well as RNA17 and RNA18 (Fig 2C). We attempted to identify a *cox3* gene using primers matching RNA8 and RNA11 sequences, which flank *cox3* in other *Babesia* s.s. and *Theileria* species (Fig 2A, S6 Table). This PCR produced a 700 base pair amplicon that contained a *cox3*-like sequence. The sequence (KF410591) had a cytochrome *c* oxidase characteristic heme-copper oxidase domain and shared 21–25% identity with *Theileria*, *Babesia*, and *Plasmodium cox3* proteins (NCBI BLAST, blastx; Simple Modular Architecture Research Tool) [74,75]. The location of this *cox3*-like gene with respect to the mitochondrial genome of *B*. *conradae* remains unknown.

### Phylogenetic relationships

**Fig 3:**
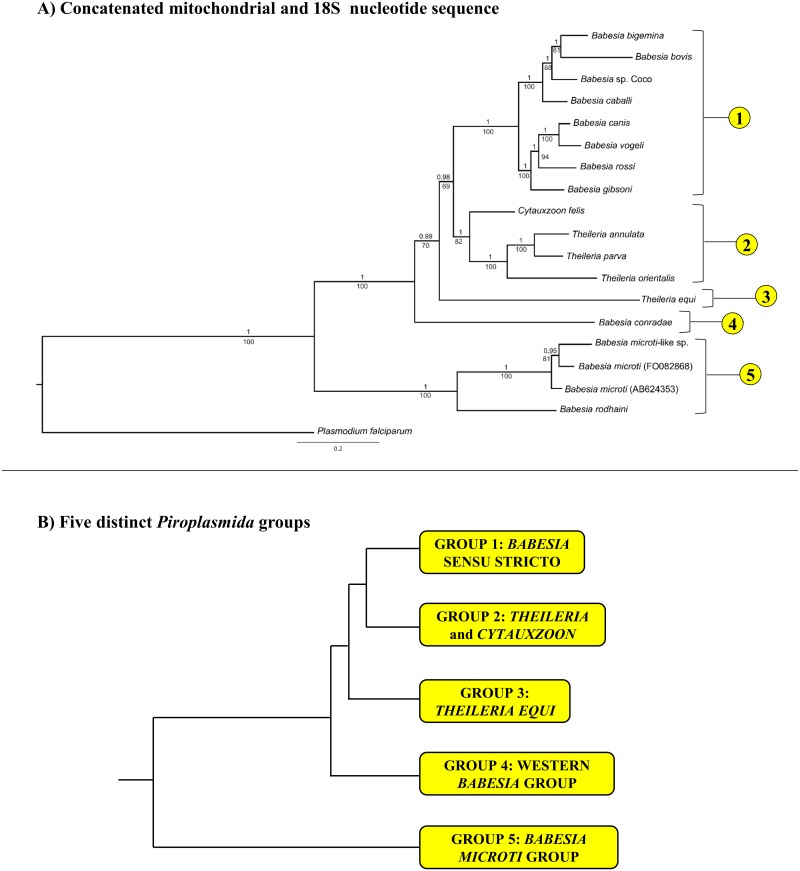
Phylogenetic analysis of concatenated mitochondrial genome and 18S nucleotide sequence identifies five distinct lineages within *Piroplasmida*. Statistical support for clades are indicated at each node: posterior probabilities from Bayesian analysis (10 million generations of Markov chain Monte Carlo) are listed above nodes, while bootstrap values from Maximum Likelihood analysis (200 bootstrap replicates) are listed below nodes. Trees are drawn to scale, with branch lengths measured in the number of substitutions per site. A) Analysis of concatenated mitochondrial and 18S nucleotide sequences (6006 total characters); see S8 Table for specific sequences included in analysis. The five lineages identified by analysis of concatenated mitochondrial and 18S nucleotide sequences are depicted in B (branch lengths not to scale).

Phylogenetic analyses of full, concatenated, mitochondrial sequences and available 18S sequences yielded a tree topology with strong statistical support (Bayesian analysis, posterior probability ≥ 0.98) at all nodes (Fig 3A). Many of the clades recovered (e.g. *Babesia* sensu stricto, Western *Babesia* group, *Babesia microti* group, and *T*. *equi*) correspond with groups described previously (Fig 1). In contrast, *Cytauxzoon felis* and *Theileria* sensu stricto species were grouped together in a single clade that is the sister group to *Babesia* sensu stricto. Results of maximum likelihood analysis reflected that of Bayesian analysis, although support for some clades (*B*. *conradae*, *T*. *equi*) was not as robust (Fig 3A).

**Fig 4:**
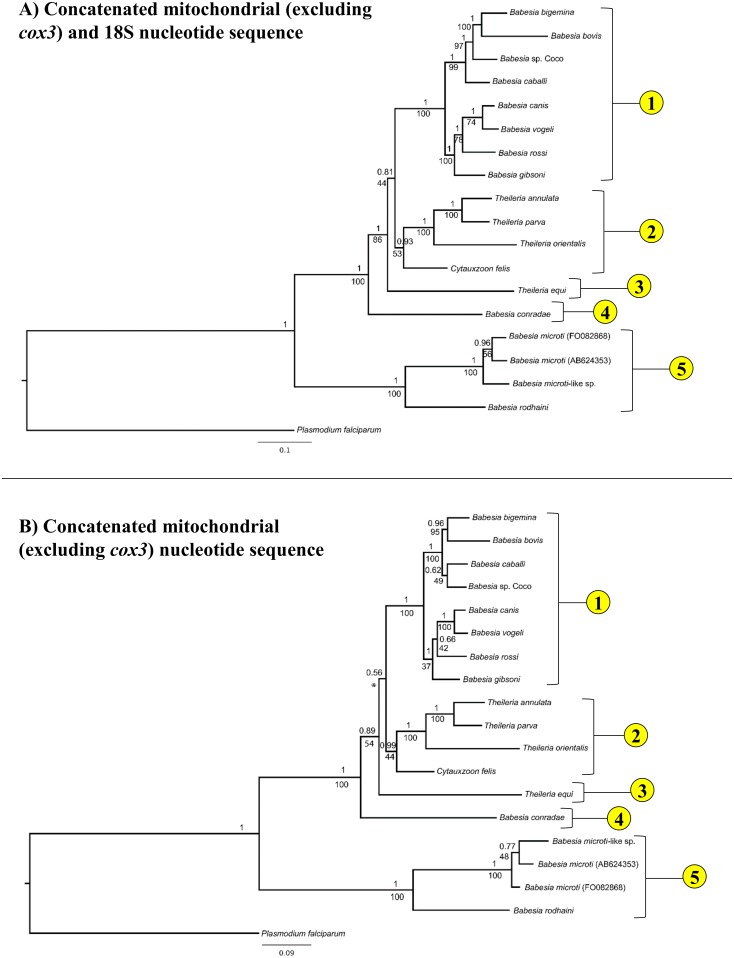
Removal of *cox3* and 18S sequence for phylogenetic analysis identifies same five distinct lineages within *Piroplasmida* but with less statistical support. Statistical support for clades are indicated at each node: posterior probabilities from Bayesian analysis (10 million generations of Markov chain Monte Carlo) are listed above nodes, while bootstrap values from Maximum Likelihood analysis (100 bootstrap replicates) are listed below nodes. Trees are drawn to scale, with branch lengths measured in the number of substitutions per site. A) Analysis of concatenated mitochondrial and 18S nucleotide sequences with *cox3* sequences excluded (5292 total characters) and B) mitochondrial nucleotide sequence alone (4395 total characters). Maximum Likelihood analysis of mitochondrial sequences alone did not recover Group 3 (*T*. *equi*) as a distinct clade, which is denoted with an asterisk (*).

**Fig 5:**
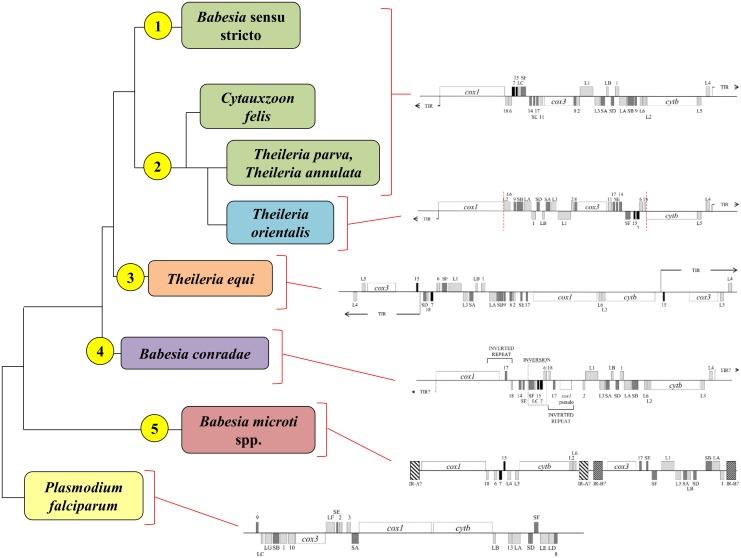
Mitochondrial genome structures further support recognition of the five groups identified by phylogenetic analysis of concatenated mitochondrial and 18S sequences. Groups are indicated by yellow circles to the left of respective clades. Genes are indicated with names (*cox1*, *cox3*, *cytb*) and ribosomal sequences are indicated in gray with “L” for large subunit and “S” for small subunit. Genes placed above the black central line on the diagram are coded on the sense strand of DNA, while those below the line are coded on the anti-sense strand. The presence or absence of TIRs in *B*. *conradae*’s mitochondrial genome has not been confirmed. Branches not drawn to scale.

**Fig 6:**
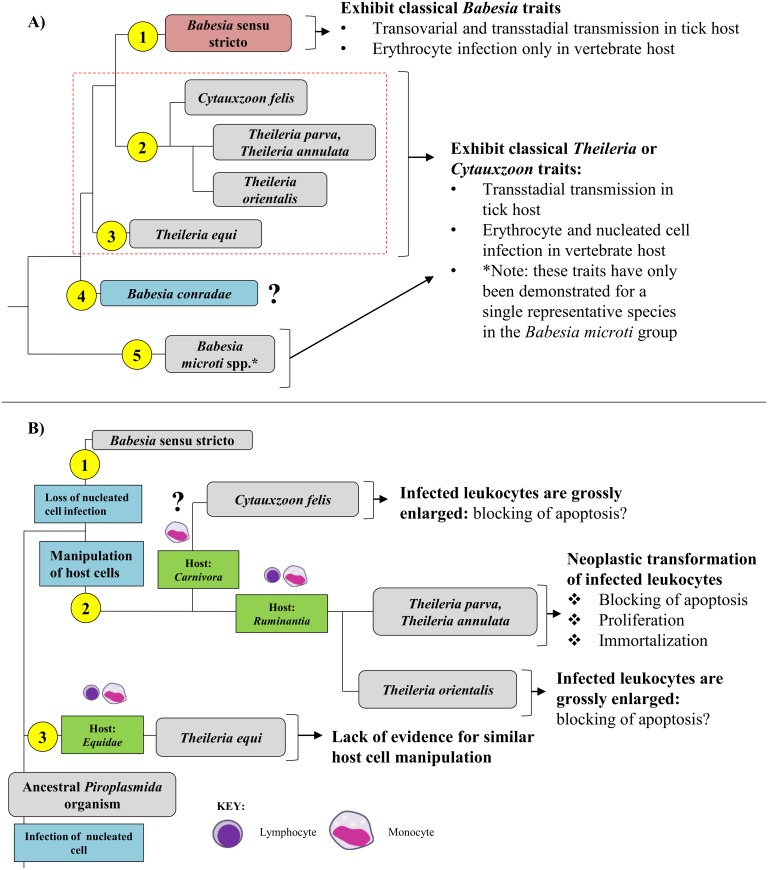
Biology of *Piroplasmida* organisms is consistent with phylogeny inferred from analysis of concatenated mitochondrial and 18S sequences. Groups are indicated by yellow circles to the left of respective clades. A) Organisms in Group 1 (*Babesia* sensu stricto; red) do not infect leukocytes and can be transmitted transovarially in the tick host, two traits unique to the group. Organisms in Groups 2, 3, and 5 (gray) are thought to be limited to transstadial transmission in the tick host and infect nucleated cells prior to erythrocytes. Notably, details regarding tick hosts, transmission in the tick, and infection of nucleated cells for Group 4 (blue) remains unknown, and infection of nucleated host cells has only been demonstrated for a single species in Group 5 (24). Characteristics of species in Groups 2 and 3 (outlined with dashed red line) are further summarized in B. B) While many organisms in Group 2 and 3 have been demonstrated to infect leukocytes, the specific leukocyte infected isn’t clade-specific and hasn’t even been confirmed for some species (e.g., *Cytauxzoon felis*). Additionally, the shared biological features of organisms in Group 2 support their distinction from the organism in Group 3, *T*. *equi*. *T*. *equi* exclusively infects equine hosts, and disease is caused by parasite infection of erythrocytes rather than the brief schizogonous phase in leukocytes. However, there is evidence indicating that organisms in Group 2 have evolved more complex methods of host leukocyte manipulation. Species within Group 2 that diverged earliest (*Cytauxzoon felis*) exclusively infect carnivores and have grossly enlarged schizont-infected cells, which suggests a blocking of host cell apoptosis. The remaining *Theileria* species in Group 2 exclusively infect ruminants. Organisms in the next clade to diverge in Group 2, including *Theileria orientalis*, also have grossly enlarged schizont-infected cells. This group is commonly known as the “non-transforming” *Theileria* species. This is in contrast to the “transforming” *Theileria* species (*T*. *annulata* and *parva*), which reversibly transform infected host leukocytes into a proliferative neoplastic state to support the replicating parasite. Branches not drawn to scale.

Because it is unclear whether *cox3*-like sequences of *T*. *equi* and *B*. *conradae* are true *cox3* orthologs, *cox3* sequences were excluded from analysis, resulting in an identical topology that had only moderate statistical support (posterior probability = 0.81, bootstrap value = 44) for the placement of *T*. *equi* but strong support for the placement of *B*. *conradae* (posterior probability = 1, bootstrap value = 86, Fig 4A). When mitochondrial sequences (excluding *cox3*) were analyzed alone, the exact placement of *T*. *equi* became unclear when comparing the two different analysis methods (posterior probability = 0.56, not recovered as separate clade by maximum likelihood), and support for the placement of *B*. *conradae* also decreased (posterior probability = 0.89, bootstrap value = 54; Fig 4B). Although bootstrap support for the placement of *C*. *felis* decreased as fewer sequences were analyzed (bootstrap value = 53 in Fig 4A, 44 in Fig 4B), more extensive Bayesian analyses consistently support this placement (posterior probability ≥ 0.93 Fig 4). All analyses strongly support (posterior probability = 1, bootstrap value = 100) that species in the *B*. *microti* group diverged at an early time point from the rest of the *Piroplasmida* (Figs 3 and 4). Therefore, our Bayesian analysis strongly supports the presence of five distinct lineages, including the *Babesia microti* group, *Babesia* sensu stricto, *Theileria* and *Cytauxzoon*, the Western *Babesia* group (represented by *B*. *conradae*), and *T*. *equi*. This phylogeny is further supported by the unique mitochondrial genome structures (Fig 5) and distinctive biological traits (Fig 6) characterizing each of the five proposed groups.

**Fig 7:**
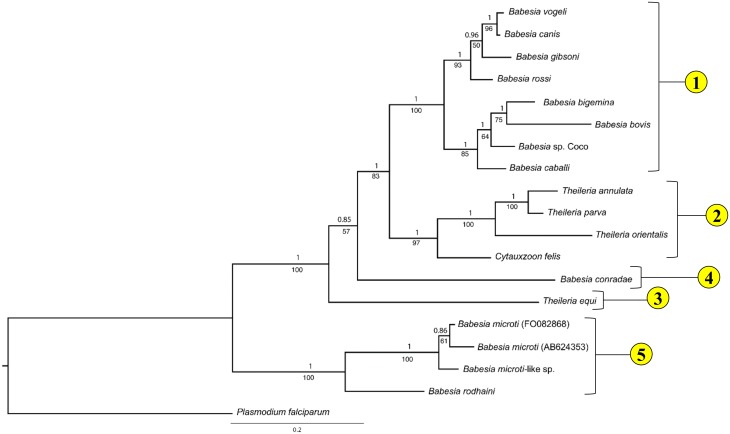
Phylogenetic analysis of *cox1* putative amino acid sequence recovers the same five *Piroplasmida* groups as concatenated mitochondrial and 18S nucleotide sequences. Statistical support for clades are indicated at each node: posterior probabilities from Bayesian analysis (10 million generations of Markov chain Monte Carlo) are listed above nodes, while bootstrap values from Maximum Likelihood analysis (100 bootstrap replicates) are listed below nodes. Tree is drawn to scale, with branch lengths measured in the number of substitutions per site; 429 characters were analyzed.

Individual mitochondrial genes were also assessed to evaluate whether these phylogenetic relationships could also be inferred from shorter, more easily obtained, mitochondrial sequences. Analysis of *cox1* amino acid sequences yields a similar topology and recovers all five lineages (Fig 7). Interestingly, in this analysis *T*. *equi* diverges prior to *B*. *conradae*, albeit with somewhat reduced statistical support (posterior probability = 0.85, bootstrap value = 57; Fig 7). Analyses of other mitochondrial genes in combination yield tree topologies that differ from the tree found by analysis of the concatenated mitochondrial and 18S sequences, but often have lower branch support at deep nodes (see S5–S9 Figs).

## Discussion

Mitochondrial genome sequences and structures provide abundant new evidence on the phylogenetic relationships of organisms in the order *Piroplasmida*. Analyses of the concatenated sequences of all identified mitochondrial genes with 18S gene sequence for only 18 species supported the existence of five distinct lineages of *Piroplasmida*. This includes four previously identified clades (*B*. *microti* group, *Babesia* sensu stricto, *Theileria equi*, and the Western *Babesia* group) and a fifth clade that includes *Theileria* sensu stricto species as well as *Cytauxzoon felis*. Recognition of these five groups is further supported by mitochondrial gene organization and biological attributes, and the groups can be easily distinguished from one another through phylogenetic analysis of *cox1* amino acid sequences alone.

Although “*Babesia*” organisms have been informally divided into species exhibiting classical *Babesia* traits (*Babesia* sensu stricto) and those that don’t (*Babesia* sensu latu), mitochondrial genome sequences and structures indicate that “*Babesia*” encompasses at least three distinct groups, which we presently refer to as the *Babesia microti* group, the Western *Babesia* group, and *Babesia* sensu stricto.

Despite including only four representatives from the clade, mitochondrial genome sequences and structures clearly support the “*Babesia microti* group” representing a distinct lineage that diverged early from the common ancestral stem of all other *Piroplasmida* (Figs 3–5 and 7). This group includes *B*. *microti*-like sp. (found in foxes and Spanish dogs), whose mitochondrial genome was first characterized in this study. Although this organism has been renamed a number of times (*Babesia microti*-like sp., *Theileria annae*, *Babesia* cf. *microti*, and most recently *Babesia vulpes*), our findings support placement in the *Babesia microti* group [76]. Furthermore, our analysis underscores the fact that this group is not only distinct, but also highly divergent from other *Piroplasmida* (Figs 3 and 7). The *Babesia microti* group is remarkable in its diversity. More than any other *Piroplasmida* clade, organisms in the *Babesia microti* group have radiated to infect a wide variety of hosts occupying a large number of niches worldwide [21,76,77]. This large group likely consists of multiple subgroups [76], and although our study lacks enough species sampling to properly address topology within the *Babesia microti* group, our phylogenetic analysis concurs with studies that subdivide *Babesia microti* organisms from *Babesia rodhaini* (Figs 3 and 7) [21,76]. Additionally, amino acid sequences of protein-coding genes was conserved (83–95% identity) between *Babesia microti*-like sp. and both *Babesia microti* isolates, but was not as well-conserved between *B*. *rodhaini* and other species in the group (50–79% identity). Nevertheless, mitochondrial genome synteny was conserved across all four species for those regions of the mitochondrial genome sequenced. Importantly, despite the apparent early divergence of the group, infection of nucleated cells in the vertebrate host has been demonstrated for only a single species in the *Babesia microti* group (Fig 6) [25]. Thus, it remains unclear whether invasion of nucleated cells is a primitive trait that has not been identified for other species, or if it is a character trait that has been gained and then lost in the evolution of the *Piroplasmida*. Regardless, the *Babesia microti* group is a well-supported lineage, and a new genus name should be strongly considered for this group to distinguish it from all other *Babesia* [43].

Similarly, analysis of concatenated mitochondrial genome and 18S sequence confirm *Babesia conradae* as a representative of a distinct lineage (Figs 3A and 4A), which we refer to as the “Western *Babesia* group.” Statistical support for this clade was robust in the majority of Bayesian analyses conducted in this study (Figs 3A, 4A and 7), but in some cases could not be confirmed by maximum likelihood analyses (Figs 3A and 4B). This can likely be attributed to the fact that only a single species from this group was represented in these analyses. Furthermore, *Babesia conradae* has a novel mitochondrial genome structure that lacks a *cox3* gene, and instead has an inverted repeat of an adjacent region of the mitochondrial genome (Figs 2 and 5). The function of this sequence is unclear, but it may play a role in facilitating recombination during mitochondrial genome replication like the internal repeats (IRs) found in the *Babesia microti* group [64]. This unique feature supports the notion of the Western *Babesia* group being distinguished from *Babesia*. We suspect that as more mitochondrial genomes are characterized for species in this group (e.g. *Babesia duncani*/WA1, *Babesia lengau*, *Babesia behnkei*), similar mitochondrial genome structures and sequences may be identified to further confirm the autonomy of this group. However, the current scarcity of information available for this group make it impossible to corroborate molecular analyses with biological data. Although our analysis and others (Fig 1) suggest that this is an older lineage that diverged after the *Babesia microti* group, these organisms have only been recognized within the last few decades [22,78–82], and many are not yet fully understood. Originally thought to be localized in the Western United States, the discovery of *Babesia lengau* and *lengau*-like species in Africa and Europe verified that organisms in this clade are distributed worldwide [79,80,82]. Currently described vertebrate hosts include ungulates, humans, and carnivores, while tick vectors remain unknown [22,78–82]. Disease severity varies between species, but is always due to intraerythrocytic organisms, as no intraleukocytic schizonts have been identified (Fig 6) [22,23]. Clearly, more studies are needed to fully understand the evolutionary relationships of this group and to verify their taxonomic placement, including characterization of additional mitochondrial genomes and, perhaps most importantly, assessment of their ability to invade nucleated cells.

Mitochondrial data also allow *Babesia conradae* to be easily distinguished from *Babesia* sensu stricto (Figs 3 and 4). When first discovered, *Babesia conradae* was identified as *Babesia gibsoni* “USA,” a nomenclature that persisted for years and continues to cause confusion in the literature [81]. However, *Babesia* sensu stricto, which includes *Babesia gibsoni*, is a monophyletic group supported by both molecular and biological data (Figs 3–6). As found previously [7,10,11], so-called large (*B*. *caballi*, *Babesia* sp. Coco, *B*. *bigemina*, *B*. *canis*, *B*. *rossi*, *B*. *vogeli*) and small (*B*. *gibsoni*, *B*. *bovis*) *Babesia* species were grouped closely together within the *Babesia* s.s. clade, confirming that piroplasm morphology is not indicative of genetic relatedness (Figs 3, 4 and 7). Rather than piroplasm morphology, it may be that host species tropism is predictive of subgrouping within *Babesia* s.s. In fact, previous studies have further subdivided this group into species that infect ungulates and species that infect carnivores [7,10]. While our limited analysis generally supports this observation, we refrain from recognizing these subgroups, as evidence is mounting to suggest that *Babesia* sensu stricto species can infect multiple vertebrate hosts [77,83–87]. Despite this, it is important to note that *Babesia* sp. Coco has only been identified in domestic dogs, yet is taxonomically grouped with species that traditionally infect ungulates. Because *Babesia* sp. Coco has primarily been identified in immunocompromised dogs [88,89], we speculate that carnivores are not the natural host and that wild ungulates should also be considered when searching for a reservoir host.

Analyses of mitochondrial genomes also indicate that reorganization of the genera currently referred to as *Theileria* and *Cytauxzoon* should be considered. The taxonomic placement of *T*. *equi* and *C*. *felis* with respect to *Theileria* species has varied in previous phylogenetic analyses of 18S alone (Fig 1; [7,9–11,30]). One study groups *T*. *equi* in the *Theileria* clade while separating *C*. *felis* in a unique clade (Fig 1A; [9]); another study classifies these species in a single clade (Fig 1B; [10]); and two other studies classify these species as three unique clades (Fig 1C and 1D; [7,11]). In contrast, our analysis of concatenated mitochondrial and 18S sequences supports the placement of *Cytauxzoon felis* and *Theileria* in a single clade that is distinct from a unique *Theileria equi* clade (Figs 3 and 4). A close taxonomic relationship between *Cytauxzoon* and these *Theileria* species is further supported by shared mitochondrial genome structures (Fig 5) as well as the unique biological features shared between the organisms (Fig 6). While previous classification schemes of *Piroplasmida* proposed recognizing groups based on the specific leukocyte infected (i.e., *Cytauxzoon* vs. *Theileria*), the discovery of *Piroplasmida* species that infect multiple leukocyte lineages indicates that such an approach is invalid (Fig 6) [26–29,44,90]. Alternatively, we propose that different *Piroplasmida* lineages may be better defined by the extent of their interaction with host cells and the strategies they employed to enhance their propagation. For instance, in contrast to all other *Piroplasmida* lineages recovered in this study, evidence of advanced host leukocyte manipulation has been observed in representative organisms from all three branches within the *Theileria/Cytauxzoon* clade (Fig 6). Unlike species outside of this clade, these organisms seem to hijack host cell functions to facilitate replication and perhaps to evade the host immune response. The most recently diverged “transforming” *Theileria* species, comprised of *T*. *annulata*, *T*. *lestoquardi*, and *T*. *parva*, have perfected this strategy by transforming host cells into a neoplastic state. Host cells transformed by these parasites are immortalized, and will evade apoptosis and proliferate as long as they remain infected [90]. Species in the older “non-transforming” *Theileria* and *Cytauxzoon* lineages do not appear to neoplastically transform host cells, but the presence of grossly enlarged schizont-infected leukocytes suggest a blocking of host cell apoptosis [90–93]. Our analysis, which includes an early divergence of *C*. *felis* and *T*. *orientalis* from the transforming *Theileria* species, corroborates these phenotypes (Fig 6). Notably, these morphological phenotypes have not been described for all species in these lineages, including the newly discovered *Cytauxzoon* species in Europe, Asia, and Africa [35–38]. However, the extent of interaction between host and parasite is likely host-specific, as evidenced by the more limited schizogony that occurs in *C*. *felis*-infected bobcats compared to in cats [94]. Thus, it is likely that other *Cytauxzoon* species infect leukocytes but that the stage isn’t recognized due to its transience in parasite-adapted hosts.

In addition to the presently described analyses, full genome data also supports the notion that transforming *Theileria* species have recently diverged from other species in the *Theileria/Cytauxzoon* clade. Parasite genes (TashAT/TpHN) that encode proteins thought to contribute to host cell transformation are present in high copy numbers (up to 20 copies) in the genomes of *T*. *annulata* and *T*. *parva* [95]. However, only a single TashAT ortholog has been identified in the genome of *T*. *orientalis* [95], and our laboratory has failed to identify any definitive orthologs in the *C*. *felis* genome (*Cytauxzoon felis* strain Winnie, http://piroplasmadb.org [96]). These data may suggest mechanisms behind the varying abilities of these species to manipulate host cells. Collectively, these observations have a number of implications for comparative studies of these organisms. For example, previously, *T*. *annulata* and *T*. *parva* had been considered the closest relatives of *C*. *felis*, and have been focused on as model species in comparative studies investigating novel therapeutic and vaccination strategies to combat cytauxzoonosis [97,98]. However, the genomic and phenotypic observations made in this studies and others [95] suggest that non-transforming *Theileria* species and *Cytauxzoon felis* may be better model species for one another, and should be taken into consideration in future comparative studies.

Additionally, in our phylogenetic analysis of concatenated mitochondrial and 18S sequences, *Theileria equi* was recovered as a unique lineage that is a sister group to the clade containing *Babesia* sensu stricto and all other *Theileria* and *Cytauxzoon* species (Fig 3). This result supports the autonomy of *T*. *equi* and confirms the placement of *T*. *equi* within *Piroplasmida* [7,42,45]. Furthermore, the mitochondrial genome structure of *T*. *equi* is radically divergent from that of any other *Piroplasmida*, which strongly supports the phylogenetic placement of *T*. *equi*. The *T*. *equi* mitochondrial genome is at least 1.5 kb longer than that of any other and possesses duplicated TIR-embedded genes, including a *cox3*-like gene [58]. This feature of long, gene-embedded TIRs is thought to be a trait possessed by the ancestor of *T*. *equi* that has been subsequently lost in later diverging species, including *Babesia* s.s., *Cytauxzoon*, and *Theileria* s.s. [58]. Characterization of the mitochondrial genome and parasite features of *Babesia bicornis*, a species grouped in the *T*. *equi* clade based on 18S analysis [7,46], will likely aid in the resolution of the evolutionary relationships of this clade. Lack of evidence for host leukocyte manipulation by *T*. *equi*, which is a feature exhibited by other *Theileria* and *Cytauxzoon* species (Fig 6), further differentiates *T*. *equi*. Collectively, these observations support the notion that *T*. *equi* is distinct from both *Theileria* and *Babesia* and that a change in nomenclature should be considered.

Although we highly recommend characterization of the full mitochondrial genome sequence and structure in addition to 18S for inferring phylogeny of *Piroplasmida*, this strategy may be cumbersome if rapid identification of novel *Piroplasmida* species is desired. Phylogenetic analysis of partial *cox1* amino acid sequences are able to recover all five groups identified in this study with relatively strong node support, albeit with a slightly altered topology (Fig 7). Therefore, in instances where analysis of the complete mitochondrial genome is impractical, amplification of *cox1* using primers listed in Table 2 is recommended.

A major limitation of this study was the number of species (n = 18) included in our analyses. Although our primary focus in this study was characterization of parasites that infect canine and feline hosts, some of these parasites (e.g. *Babesia felis*, *Babesia lengau*, *Rangelia vitalli*) were not available to our laboratory. Additionally, a limited sample size is problematic when a clade is only represented by a single species, as was the case with *T*. *equi* and *B*. *conradae*. We hypothesize that support for these clades will increase as additional related species are analyzed. Similarly, having more representative species of the *Theileria/Cytauxzoon* and *Babesia microti* groups will likely aid in refining their clade taxonomy. This analysis would have benefitted from inclusion of other unique species whose phylogeny is currently unclear but may represent novel lineages, such as *Rangelia vitalli* that infects canids [99], *Theileria ornithorhynchi* in platypuses [45], or avian-infecting species such as *Babesia bennetti* or *Babesia poelea* [100,101]. However, none of these samples were available to our laboratory. As more species’ mitochondrial genomes are characterized, we anticipate that additional clades may be discovered and existing lineages will be further refined.

Unfortunately, many species within *Piroplasmida* are currently named based on vertebrate host, parasite morphology and host cell tropism, yet lack any molecular characterization. In fact, over 80% of historically described *Piroplasmida* species [33] lack publically documented molecular characterization (NCBI GenBank). The absence of molecular characterization of these species makes it very difficult to adequately resolve historically assigned nomenclature with the *Piroplasmida* clades identified by molecular phylogenetics in this studies and others. The currently assigned nomenclature obviously deserves strong reconsideration, and some clades are likely to require changes in nomenclature at the genus level. For example, in addition to being genetically divergent, organisms classified within the *B*. *microti* group display phenotypic characteristics that are inconsistent with the defining features of the genus *Babesia*, such as the infection of lymphocytes and lack of transovarial transmission [43]. Because of discrepancies such as these, the authors suggest prudence in naming new species or redefining the species nomenclature until previously designated species have adequate molecular data submitted to publically available databases and a comprehensive review and revision of the nomenclature within the order *Piroplasmida* is performed. Proposals to rename currently recognized genera and species one at a time are likely to perpetuate the confusion in the literature that is already common. The authors believe that the absence of a provisional "Candidatus" status for eukaryotes comparable to that used for nomenclature in prokaryotes (International Committee on Systematics of Prokaryotes) is a barrier for apicomplexan nomenclature [102].

## Conclusions

In conclusion, in this study we characterized seven new *Piroplasmida* mitochondrial genomes, one of which (*B*. *conradae*) possesses a novel arrangement of mitochondrial genes. When analyzing concatenated mitochondrial gene and 18S nucleotide sequences, we recovered five distinct groups of *Piroplasmida* species. While the phylogeny derived from this analysis is further supported by both mitochondrial genome structures and known biological features of the groups, more work needs to be done in characterizing the latter for many *Piroplasmida* species. In addition to 18S rRNA, future phylogenetic studies of *Piroplasmida* should include mitochondrial genome sequences and structures, or at the very least *cox1* sequence. Importantly, the results of this study clarify some taxonomic relationships and continue to raise questions about some of the nomenclature within *Piroplasmida*.

## Supporting Information

S1 Fig Schematic of PCR amplification of *B*. *canis*, *B*. *rossi*, and *C*. *felis* mitochondrial genomes. Primers were designed to amplify near full length mitochondrial genomes in three overlapping fragments. Primers for fragments 1–3 and TIRs are indicated with arrows (forward primers: F1-F3, reverse primers: R1-R3, TIR primers: TIR F/R). Genes shown above the central line are coded on the sense strand, while those below are on the antisense strand. Protein-coding genes (*cox1*, *cox3*, and *cytb*) are indicated in white. Large subunit rRNA fragments are in light gray, small subunit rRNA fragments are in dark gray, and miscellaneous conserved RNA fragments are in black.
(PDF)

S2 Fig Schematic of PCR amplification of *B*. *vogeli* and *Babesia sp*. Coco mitochondrial genomes. Because attempts at TIR amplifications were unsuccessful, primers were designed to amplify near full length mitochondrial genomes of species in five overlapping fragments. Primers for fragments 0–4 are indicated with arrows (forward primers: F0-F4, reverse primers: R0-R4). Protein-coding genes (*cox1*, *cox3*, and *cytb*) are indicated in white. Large subunit rRNA fragments are in light gray, small subunit rRNA fragments are in dark gray, and miscellaneous conserved RNA fragments are in black.
(PDF)

S3 Fig Schematic of PCR amplification of *B*. *conradae* mitochondrial genome. Because initial attempts at amplifying near full length mitochondrial genome was unsuccessful, primers were designed to amplify near full length *B*. *conradae* mitochondrial genome in six overlapping fragments. Primers for fragments 0–4 are indicated with arrows (forward primers: F0-F4, reverse primers: R0-R4). Protein-coding genes (*cox1*, *cox3*, and *cytb*) are indicated in white. Large subunit rRNA fragments are in light gray, small subunit rRNA fragments are in dark gray, and miscellaneous conserved RNA fragments are in black.
(PDF)

S4 Fig Schematic of PCR amplification of *B*. *microti*-like sp. mitochondrial genome. Because of the mitochondrial genome structure of close relative *B*. *microti* is known to differ from that of *Babesia* sensu stricto species, an alternative approach was employed to amplify the *B*. *microti*-like sp. mitochondrial genome. Primers were designed to amplify near full length mitochondrial genomes of species in six fragments that formed two separate contigs. Amplification across assumed inverted repeats (indicated by IR-A and IR-B) was not pursued due to lack of informative phylogenetic sequence in this region; hence, a single contig of the mitochondrial genome was not obtained. Primers for fragments 1–6 are indicated with arrows (forward primers: F1-F6, reverse primers: R1-R6). Protein-coding genes (*cox1*, *cox3*, and *cytb*) are indicated in white. Large subunit rRNA fragments are in light gray, small subunit rRNA fragments are in dark gray, and miscellaneous conserved RNA fragments are in black.
(PDF)

S5 Fig Phylogenetic analysis of *cox1* nucleotide sequence does not recover the five distinct lineages identified by analysis of concatenated mitochondrial and 18S sequences. Statistical support for clades are indicated at each node: posterior probabilities from Bayesian analysis (10 million generations of Markov chain Monte Carlo) are listed above nodes, while bootstrap values from Maximum Likelihood analysis (100 bootstrap replicates) are listed below nodes. Tree is drawn to scale, with branch lengths measured in the number of substitutions per site. Analysis of 1287 total characters produced a tree with decent statistical support, but the *Babesia conradae* clade was grouped within the *Theileria* clade, which does not reflect the results of analysis of concatenated mitochondrial and 18S sequences.
(PDF)

S6 Fig Phylogenetic analysis of *cytb* nucleotide sequence does not recover the five distinct lineages identified by analysis of concatenated mitochondrial and 18S sequences. Statistical support for clades are indicated at each node: posterior probabilities from Bayesian analysis (10 million generations of Markov chain Monte Carlo) are listed above nodes, while bootstrap values from Maximum Likelihood analysis (100 bootstrap replicates) are listed below nodes. Tree is drawn to scale, with branch lengths measured in the number of substitutions per site. Analysis of 1095 total characters produced a tree with poor statistical support for many nodes and a topology that does not reflect the results of analysis of concatenated mitochondrial and 18S sequences.
(PDF)

S7 Fig Phylogenetic analysis of *cytb* amino acid sequence does not recover the five distinct lineages identified by analysis of concatenated mitochondrial and 18S sequences. Statistical support for clades (bootstrap values from Maximum Likelihood analysis, 100 bootstrap replicates) are indicated below each node. Tree is drawn to scale, with branch lengths measured in the number of substitutions per site. Analysis of 365 total characters produced a tree with poor statistical support for many nodes and a topology that does not reflect the results of analysis of concatenated mitochondrial and 18S sequences.
(PDF)

S8 Fig Phylogenetic analysis of concatenated *cox1* and *cytb* nucleotide sequence does not recover the five distinct lineages identified by analysis of concatenated mitochondrial and 18S sequences. Statistical support for clades are indicated at each node: posterior probabilities from Bayesian analysis (10 million generations of Markov chain Monte Carlo) are listed above nodes, while bootstrap values from Maximum Likelihood analysis (100 bootstrap replicates) are listed below nodes. Tree is drawn to scale, with branch lengths measured in the number of substitutions per site. Analysis of 2382 total characters produced a tree with poor statistical support for multiple nodes and a topology that does not reflect the results of analysis of concatenated mitochondrial and 18S sequences.
(PDF)

S9 Fig Phylogenetic analysis of concatenated *cox1* and *cytb* amino acid sequence recovers the five distinct lineages identified by analysis of concatenated mitochondrial and 18S sequences but with poorer statistical support than analysis *cox1* amino acid alone. Statistical support for clades (bootstrap values from Maximum Likelihood analysis, 100 bootstrap replicates) are indicated below each node. Tree is drawn to scale, with branch lengths measured in the number of substitutions per site. Analysis of 794 total characters produced a tree with topology identical to that of analysis of *cox1* amino acid sequence, but had poorer statistical support.
(PDF)

S1 Table Primers utilized in additional *C*. *felis* PCR assays. (PDF)

S2 Table Primers utilized in additional *B*. *canis* PCR assays. (PDF)

S3 Table Primers utilized in additional *B*. *rossi* PCR assays. (PDF)

S4 Table Primers utilized in additional *B*. *vogeli* PCR assays. (PDF)

S5 Table Primers utilized in additional *Babesia* sp. Coco PCR assays. (PDF)

S6 Table Primers utilized in additional *B*. *conradae* PCR assays. (PDF)

S7 Table Primers utilized in additional *B*. *microti*-like sp. PCR assays. (PDF)

S8 Table Locations of protein-coding genes and rRNA fragments within *Piroplasmida* mitochondrial genomes. Gene/fragment coordinates with a white background are coded on the sense strand, while those highlighted in yellow are on the antisense strand. Some genes/fragments were not identified in some mitochondrial genomes (indicated in green and with “No ID”) while others were duplicated for some mitochondrial genomes (indicated in blue). Coordinates initially reported in separate studies are not reported in this table (noted in gray, “previously published”). Genes/fragments that were identified for all species were included in phylogenetic analysis (highlighted in red).
(PDF)
