# Supplementary material for: Mitochondrial Genome Sequences and Structures Aid in the Resolution of Piroplasmida phylogeny
Source: PLoS One. 2016 Nov 10;11(11):e0165702. doi: 10.1371/journal.pone.0165702 (PMC5104439; doi:10.1371/journal.pone.0165702)
Supplement: S1 Fig — Primers were designed to amplify near full length mitochondrial genomes in three overlapping fragments. Primers for fragments 1–3 and TIRs are indicated with arrows (forward primers: F1-F3, reverse primers: R1-R3, TIR primers: TIR F/R). Genes shown above the central line are coded on the sense strand, while those below are on the antisense strand. Protein-coding genes (cox1, cox3, and cytb) are indicated in white. Large subunit rRNA fragments are in light gray, small subunit rRNA fragments are in dark gray, and miscellaneous conserved RNA fragments are in black. (PDF) [file pone.0165702.s001.pdf]

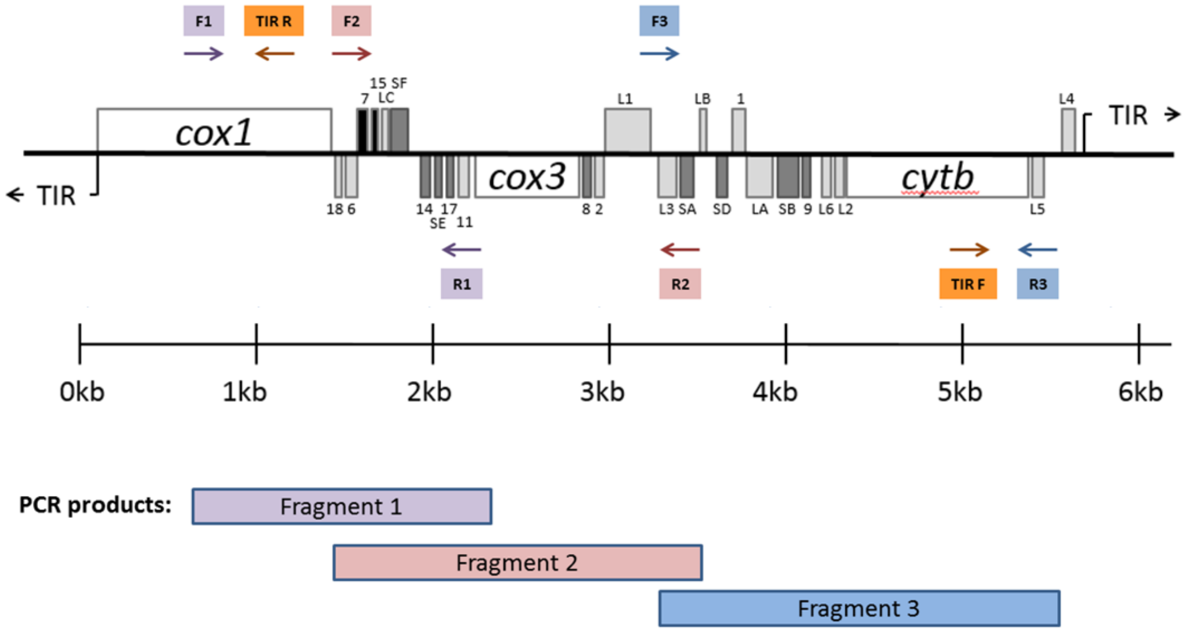

S1 Figure. Schematic of PCR amplification of *B. canis*, *B. rossi*, and *C. felis* mitochondrial genomes. Primers were designed to amplify near full length mitochondrial genomes in three overlapping fragments. Primers for fragments 1-3 and TIRs are indicated with arrows (forward primers: F1-F3, reverse primers: R1-R3, TIR primers: TIR F/R). Genes shown above the central line are coded on the sense strand, while those below are on the antisense strand. Protein-coding genes (*cox1*, *cox3*, and *cytb*) are indicated in white. Large subunit rRNA fragments are in light gray, small subunit rRNA fragments are in dark gray, and miscellaneous conserved RNA fragments are in black.
