# Supplementary material for: Mitochondrial Genome Sequences and Structures Aid in the Resolution of Piroplasmida phylogeny
Source: PLoS One. 2016 Nov 10;11(11):e0165702. doi: 10.1371/journal.pone.0165702 (PMC5104439; doi:10.1371/journal.pone.0165702)
Supplement: S3 Fig — Because initial attempts at amplifying near full length mitochondrial genome was unsuccessful, primers were designed to amplify near full length B. conradae mitochondrial genome in six overlapping fragments. Primers for fragments 0–4 are indicated with arrows (forward primers: F0-F4, reverse primers: R0-R4). Protein-coding genes (cox1, cox3, and cytb) are indicated in white. Large subunit rRNA fragments are in light gray, small subunit rRNA fragments are in dark gray, and miscellaneous conserved RNA fragments are in black. (PDF) [file pone.0165702.s003.pdf]

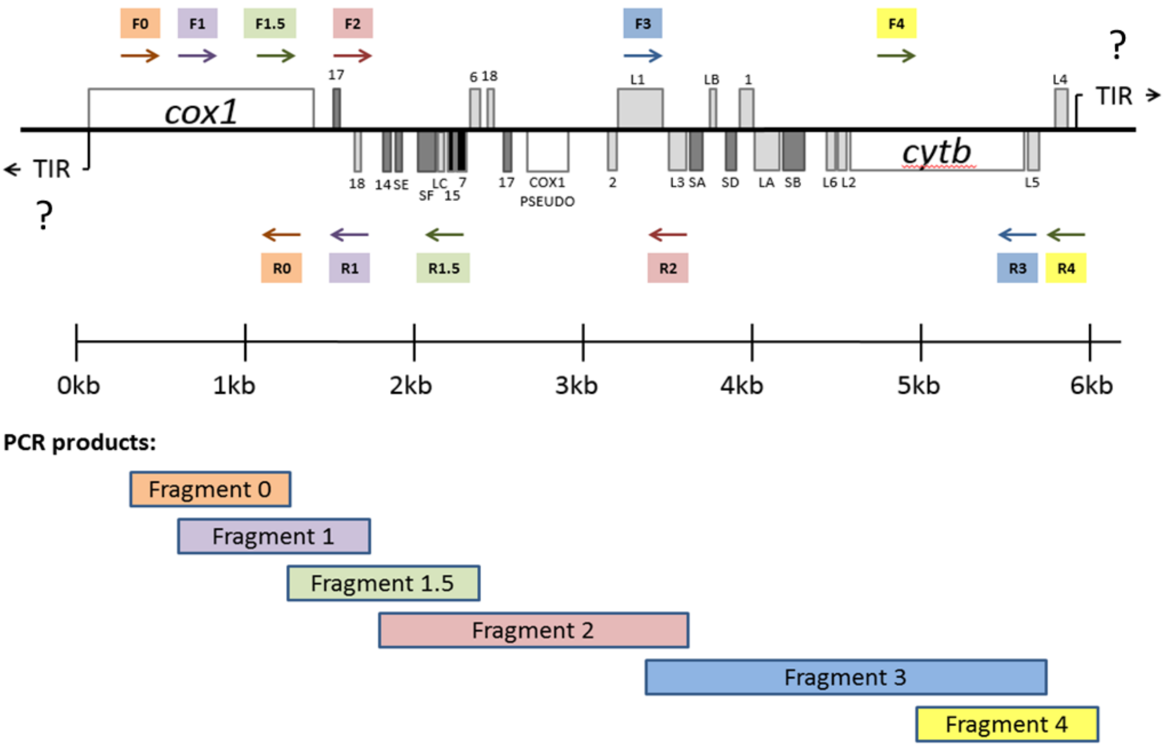


**S3 Figure. Schematic of PCR amplification of *B. conradae* mitochondrial genome.** Because initial attempts at amplifying near full length mitochondrial genome was unsuccessful, primers were designed to amplify near full length *B. conradae* mitochondrial genome in six overlapping fragments. Primers for fragments 0-4 are indicated with arrows (forward primers: F0-F4, reverse primers: R0-R4). Protein-coding genes (*cox1*, *cox3*, and *cytb*) are indicated in white. Large subunit rRNA fragments are in light gray, small subunit rRNA fragments are in dark gray, and miscellaneous conserved RNA fragments are in black.
