# Supplementary material for: Mitochondrial Genome Sequences and Structures Aid in the Resolution of Piroplasmida phylogeny
Source: PLoS One. 2016 Nov 10;11(11):e0165702. doi: 10.1371/journal.pone.0165702 (PMC5104439; doi:10.1371/journal.pone.0165702)
Supplement: S4 Fig — Because of the mitochondrial genome structure of close relative B. microti is known to differ from that of Babesia sensu stricto species, an alternative approach was employed to amplify the B. microti-like sp. mitochondrial genome. Primers were designed to amplify near full length mitochondrial genomes of species in six fragments that formed two separate contigs. Amplification across assumed inverted repeats (indicated by IR-A and IR-B) was not pursued due to lack of informative phylogenetic sequence in this region; hence, a single contig of the mitochondrial genome was not obtained. Primers for fragments 1–6 are indicated with arrows (forward primers: F1-F6, reverse primers: R1-R6). Protein-coding genes (cox1, cox3, and cytb) are indicated in white. Large subunit rRNA fragments are in light gray, small subunit rRNA fragments are in dark gray, and miscellaneous conserved RNA fragments are in black. (PDF) [file pone.0165702.s004.pdf]

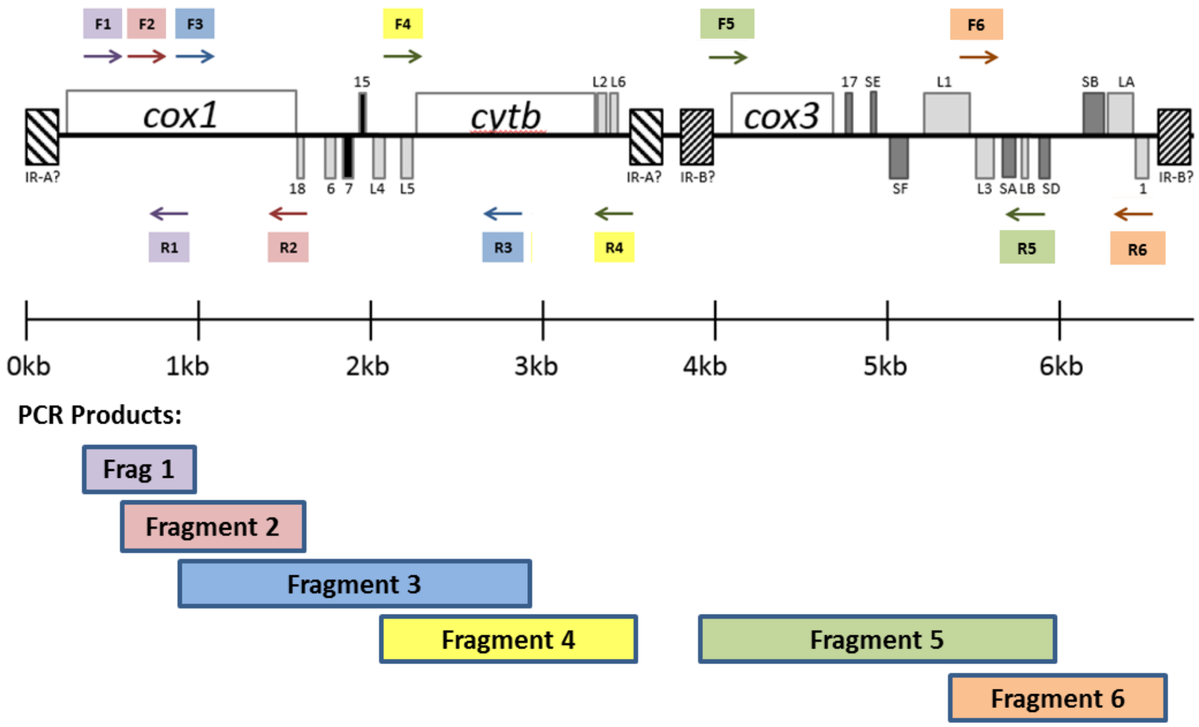

S4 Figure. Schematic of PCR amplification of *B. microti*-like sp. mitochondrial genome. Because of the mitochondrial genome structure of close relative *B. microti* is known to differ from that of *Babesia* sensu stricto species, an alternative approach was employed to amplify the *B. microti*-like sp. mitochondrial genome. Primers were designed to amplify near full length mitochondrial genomes of species in six fragments that formed two separate contigs. Amplification across assumed inverted repeats (indicated by IR-A and IR-B) was not pursued due to lack of informative phylogenetic sequence in this region; hence, a single contig of the mitochondrial genome was not obtained. Primers for fragments 1-6 are indicated with arrows (forward primers: F1-F6, reverse primers: R1-R6). Protein-coding genes (*cox1*, *cox3*, and *cytb*) are indicated in white. Large subunit rRNA fragments are in light gray, small subunit rRNA fragments are in dark gray, and miscellaneous conserved RNA fragments are in black.
