# Supplementary material for: Mitochondrial Genome Sequences and Structures Aid in the Resolution of Piroplasmida phylogeny
Source: PLoS One. 2016 Nov 10;11(11):e0165702. doi: 10.1371/journal.pone.0165702 (PMC5104439; doi:10.1371/journal.pone.0165702)
Supplement: S6 Fig — Statistical support for clades are indicated at each node: posterior probabilities from Bayesian analysis (10 million generations of Markov chain Monte Carlo) are listed above nodes, while bootstrap values from Maximum Likelihood analysis (100 bootstrap replicates) are listed below nodes. Tree is drawn to scale, with branch lengths measured in the number of substitutions per site. Analysis of 1095 total characters produced a tree with poor statistical support for many nodes and a topology that does not reflect the results of analysis of concatenated mitochondrial and 18S sequences. (PDF) [file pone.0165702.s006.pdf]

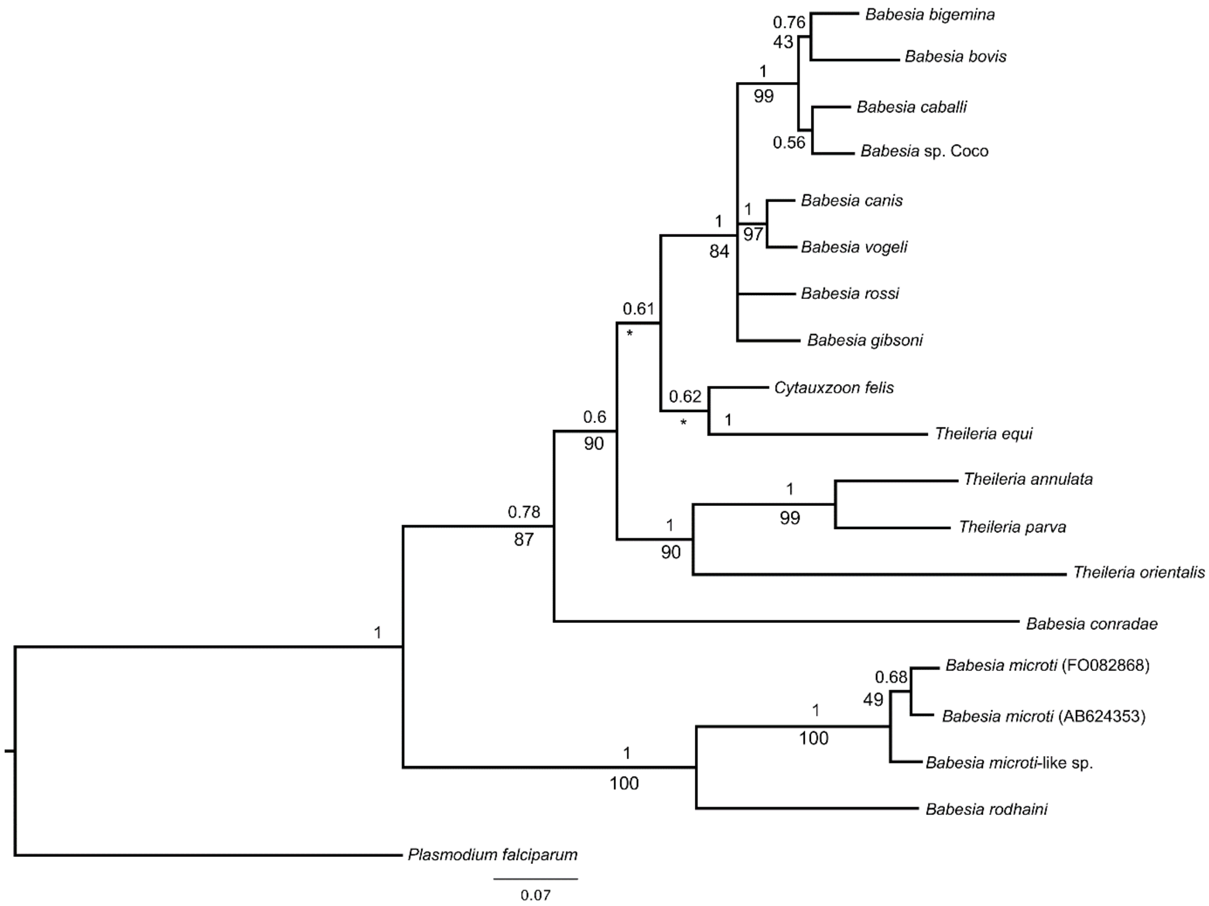

S6 Figure. Phylogenetic analysis of *cytb* nucleotide sequence does not recover the five distinct lineages identified by analysis of concatenated mitochondrial and 18S sequences. Statistical support for clades are indicated at each node: posterior probabilities from Bayesian analysis (10 million generations of Markov chain Monte Carlo) are listed above nodes, while bootstrap values from Maximum Likelihood analysis (100 bootstrap replicates) are listed below nodes. Tree is drawn to scale, with branch lengths measured in the number of substitutions per site. Analysis of 1095 total characters produced a tree with poor statistical support for many nodes and a topology that does not reflect the results of analysis of concatenated mitochondrial and 18S sequences.
