# Supplementary material for: Mitochondrial Genome Sequences and Structures Aid in the Resolution of Piroplasmida phylogeny
Source: PLoS One. 2016 Nov 10;11(11):e0165702. doi: 10.1371/journal.pone.0165702 (PMC5104439; doi:10.1371/journal.pone.0165702)
Supplement: S9 Fig — Statistical support for clades (bootstrap values from Maximum Likelihood analysis, 100 bootstrap replicates) are indicated below each node. Tree is drawn to scale, with branch lengths measured in the number of substitutions per site. Analysis of 794 total characters produced a tree with topology identical to that of analysis of cox1 amino acid sequence, but had poorer statistical support. (PDF) [file pone.0165702.s009.pdf]

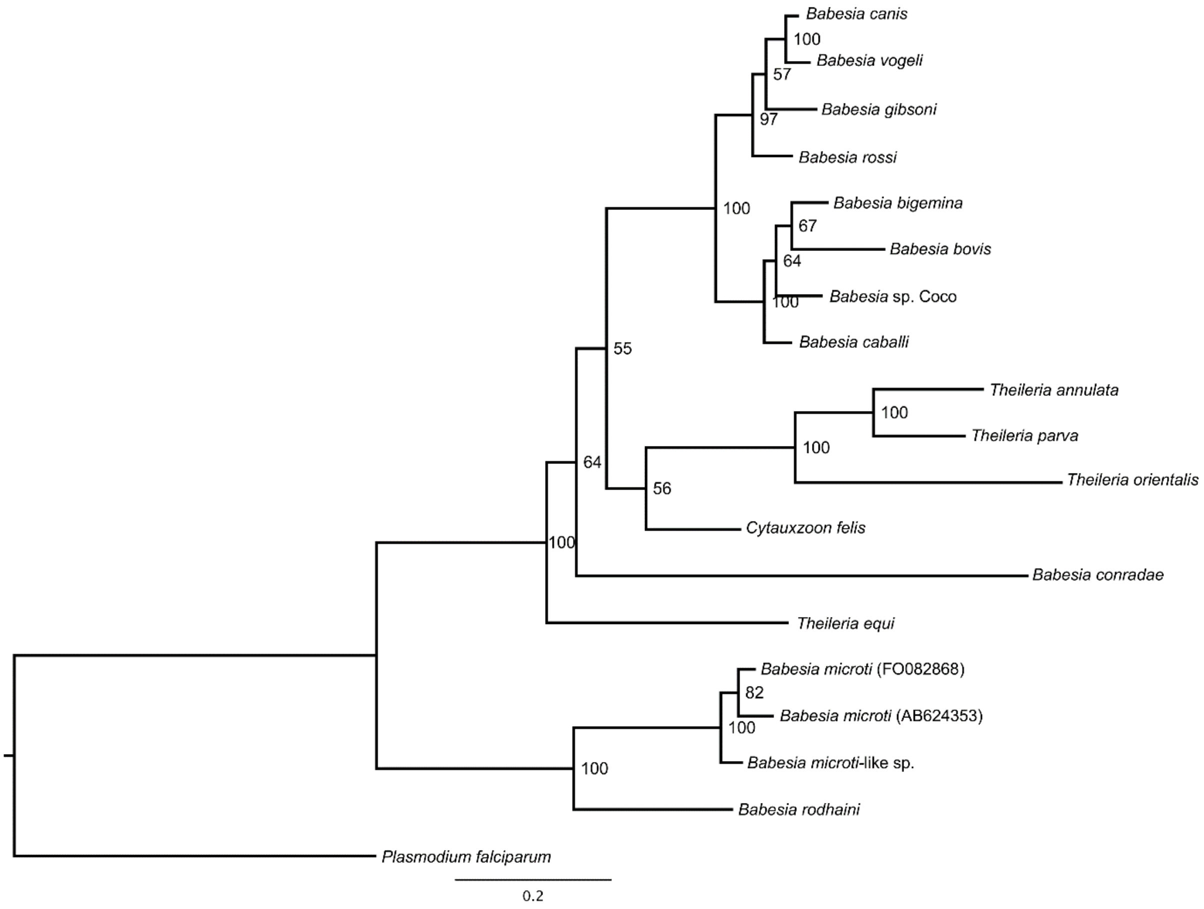

S9 Figure. Phylogenetic analysis of concatenated *cox1* and *cytb* amino acid sequence recovers the five distinct lineages identified by analysis of concatenated mitochondrial and 18S sequences but with poorer statistical support than analysis *cox1* amino acid alone. Statistical support for clades (bootstrap values from Maximum Likelihood analysis, 100 bootstrap replicates) are indicated below each node. Tree is drawn to scale, with branch lengths measured in the number of substitutions per site. Analysis of 794 total characters produced a tree with topology identical to that of analysis of *cox1* amino acid sequence, but had poorer statistical support.
