# Supplementary material for: Mitochondrial Genome Sequences and Structures Aid in the Resolution of Piroplasmida phylogeny
Source: PLoS One. 2016 Nov 10;11(11):e0165702. doi: 10.1371/journal.pone.0165702 (PMC5104439; doi:10.1371/journal.pone.0165702)
Supplement: S1 Table — (PDF) [file pone.0165702.s010.pdf]

S1 Table. Primers utilized in additional *C. felis* PCR assays.

**Purpose**	**Sequence**	**Amplicon**[c]
**Inverted PCR**[a]	ATACCTGTCAAGTTCCTTCACTA	TIR F
	TTGACACTACTAAAAGCACTAGC	TIR R
**Additional Sequencing**[b]	CAATGTGTAGAATCAGTTAGATCATTGG	Fragment 1 (F)
	ACGTATCAATATTCTCTACTCTGTTACC	Fragment 2 (F)
	ACTGAATATAATATCGCAGTAGAAGTAG	Fragment 2 (F)
	CAAGAGTGTAAGGTTCATACCATAG	Fragment 3 (F)
	CTGATAAATTGGATAATTCTGACTTAGTG	Fragment 3 (F)
	ATCTAGTGACAAGATATGAATCAACAC	Fragment 3 (F)
	CATTGAGCATGG AAATAACGGAATG	Fragment 3 (F)
	TATTCCTATAATTGCACCTATAGATAACAC	Fragment 1 (R)
	GGGATATTTACATTCTAGATGGTCAAAT	Fragment 2 (R)
	AGGAATTGATATTAGACGTCAGAAAGTAC	Fragment 3 (R)
	TATCATGTGTTGATTCATATCTTGTCAC	Fragment 3 (R)

[a]Primers were designed to amplify terminal inverted repeats (TIR); cloning was required for full sequence resolution

[b]Additional primers were designed to obtain complete bi-directional sequencing of mitochondrial fragments.

[c](F)=Forward Primer, (R)=Reverse Primer
