# Supplementary material for: Mitochondrial Genome Sequences and Structures Aid in the Resolution of Piroplasmida phylogeny
Source: PLoS One. 2016 Nov 10;11(11):e0165702. doi: 10.1371/journal.pone.0165702 (PMC5104439; doi:10.1371/journal.pone.0165702)
Supplement: S2 Table — (PDF) [file pone.0165702.s011.pdf]

S2 Table. Primers utilized in additional *B. canis* PCR assays.

**Purpose**	**Sequence**	**Amplicon**[c]
**Inverted PCR**[a]	TACCTGTCAAATTCCTTCACTAAC	TIR F
	TTGCTCCACTCATTGCAC	TIR R
**Internal Sequencing**[b]	ACA ACTGGAGTTATATTAGGAAATGC	Fragment 1 (F)
	ATGGAATCAGTATATTCCAGGGTATC	Fragment 2 (F)
	CTGATAAATTGGATAATTCTGACTTAGTG	Fragment 3 (F)
	GAATCAAATTAA ACAACATGTTCCACTG	Fragment 3 (F)
	GAATCAATTCCAGATAATGGATTAGTACT	Fragment 3 (F)
	ATACACATTGTGCATGGAAGTAACG	Fragment 3 (F)
	TCCTAAGAAATGCATTGGAATGAATG	Fragment 1 (R)
	ACGTATCAATATTCTCTACTCTGTTACC	Fragment 2 (R)
	TATGGGCTTTTTTGCATATGAGATG	Fragment 2 (R)
	CAAATGAGTTATTGGGGAGC	Fragment 3 (R)
	GGAATAGGAAAGATTAACCGCTATC	Fragment 3 (R)

[a]Primers were designed to amplify terminal inverted repeats (TIR); cloning was required for full sequence resolution

[b]Additional primers were designed to obtain complete bi-directional sequencing of mitochondrial fragments.

[c](F)=Forward Primer, (R)=Reverse Primer
