# Supplementary material for: Mitochondrial Genome Sequences and Structures Aid in the Resolution of Piroplasmida phylogeny
Source: PLoS One. 2016 Nov 10;11(11):e0165702. doi: 10.1371/journal.pone.0165702 (PMC5104439; doi:10.1371/journal.pone.0165702)
Supplement: S4 Table — (PDF) [file pone.0165702.s013.pdf]

**S4 Table. Primers utilized in additional *B. vogeli* PCR assays**.

| Purpose | Sequence | Amplicon[c] |
|---|---|---|
| **Additional mitochondrial genome PCR amplification**[a] | AGAACAGAATTGAGTATGAGTG | Fragment 0 (F) [d] |
| | CACTCATAGCACTAGCTATTCC | Fragment 0 (R) [d] |
| | CTCATTGTGCATGAAAATAGCG | Fragment 4 (F) |
| | ATACTAGATAGGGAACGAACTGC | Fragment 4 (R) |
| **Additional Sequencing**[b] | ACAACTGGAGTTATATTAGGAAATGC | Fragment 1 (F) |
| | GCGAATAACGTAGATGAGTTAGTTG | Fragment 2 (F) |
| | ACGTATCAATATTCTCTACTCTGTTACC | Fragment 2 (F) |
| | GAATCAAATTAAACAACATGTTCCACTG | Fragment 3 (F) |
| | TGTAAGCAAATACCATTCTGGTAC | Fragment 3 (F) |
| | CTCATTGTGCATGAAAATAGCG | Fragment 3 (F) |
| | TCCTAAGAAATGCATTGGAATGAATG | Fragment 1 (R) |
| | ACGTTACAGGTACTTTAAACGTAG | Fragment 2 (R) |
| | GGAATAGGAAAGATTAACCGCTATC | Fragment 3 (R) |
| | CAAATGAGTTATTGGGGAGC | Fragment 3 (R) |

[a]*B. vogeli* required alternative PCR assays to obtain additional mitochondrial genome sequence as TIR PCR attempts were unsuccessful

[b]Additional primers were designed to obtain complete bi-directional sequencing of mitochondrial fragments.

[c](F)=Forward Primer, (R)=Reverse Primer

[d]PCR amplicons required cloning for full sequence resolution
