# Supplementary material for: Mitochondrial Genome Sequences and Structures Aid in the Resolution of Piroplasmida phylogeny
Source: PLoS One. 2016 Nov 10;11(11):e0165702. doi: 10.1371/journal.pone.0165702 (PMC5104439; doi:10.1371/journal.pone.0165702)
Supplement: S5 Table — (PDF) [file pone.0165702.s014.pdf]

S5 Table. Primers utilized in additional *Babesia* sp. Coco PCR assays

Purpose	Sequence	Amplicon[c]
**Additional mitochondrial genome PCR amplification[a]**	AGAACTGAATTAAGCATGAGTG	Fragment 0 (F)
	TCCTAAGAAATGCATTGGAATGAATG	Fragment 0 (R)
	GAATCAATTCCAGATAATGGATTAGTACT	Fragment 4 (F)
	ATACTAGATAGGGAACGAACTGC	Fragment 4 (R)
**Additional Sequencing[b]**	CAATTCCACCAAAAAGTCCAGTC	Fragment 0 (R)
	CATTTTCACTTTGTTCTATCAATTGGAGC	Fragment 1 (F)
	ATGGAATCAGTATATTCCAGGGTATC	Fragment 2 (F)
	ACGTATCAATATTCTCTACTCTGTTACC	Fragment 2 (F)
	GAATCAAATTAAACAACATGTTCCACTG	Fragment 3 (F)
	CTGATAAATTGGATAATTCTGACTTAGTG	Fragment 3 (F)
	CATTGAGCATGGAAATAACGGAATG	Fragment 3 (F)
	GAATCAATTCCAGATAATGGATTAGTACT	Fragment 3 (F)
	TCCTAAGAAATGCATTGGAATGAATG	Fragment 1 (R)
	ACGTTACAGGTACTTTAAACGTAG	Fragment 2 (R)
	CAAATGAGTTATTGGGGAGC	Fragment 3 (R)
	GGAATAGGAAAGATTAACCGCTATC	Fragment 3 (R)

[a]*Babesia* sp. Coco required alternative PCR assays to obtain additional mitochondrial genome sequence as TIR PCR attempts were unsuccessful

[b]Additional primers were designed to obtain complete bi-directional sequencing of mitochondrial fragments.

[c](F)=Forward Primer, (R)=Reverse Primer
