# Supplementary material for: Mitochondrial Genome Sequences and Structures Aid in the Resolution of Piroplasmida phylogeny
Source: PLoS One. 2016 Nov 10;11(11):e0165702. doi: 10.1371/journal.pone.0165702 (PMC5104439; doi:10.1371/journal.pone.0165702)
Supplement: S6 Table — (PDF) [file pone.0165702.s015.pdf]

S6 Table. Primers utilized in additional *B. conradae* PCR assays

**Purpose**	**Sequence**	**Amplicon**[d]
**Additional mitochondrial genome PCR amplification**[a]	AGAACAGAATTGAGTATGAGTG	Fragment 0 (F) [e]
	TCCTAATATAACTCCAGTAGTACCTCC	Fragment 0 (R) [e]
	ACAACTGGAGTTATATTAGGAAATGC	Fragment 1.5 (F)
	GACAGTGAACTTGTAGCTGAAC	Fragment 1.5 (R)
	ATACGTGCCGAGCAGTGTG	Fragment 2 (F)
	CCTTGAAATCCAGGAATAATACC	Fragment 4 (F)
	ATACTAGATAGGGAACGAACTGC	Fragment 4 (R)
**Additional Sequencing**[b]	CTCTATCAACTGATAAACCAACAGATC	Fragment 0 (R)
	ACAACTGGAGTTATATTAGGAAATGC	Fragment 1 (F)
	ACATCATTGTAGCTCCTACTGTAG	Fragment 2 (F)
	AATGAGGAGCGTCTGT	Fragment 2 (F)
	CTTCGCATTAAACAGTAGGTTCC	Fragment 3 (F)
	GATACACATTGAGCATGAAAATAACG	Fragment 3 (F)
	CCTTGAAATCCAGGAATAATACC	Fragment 3 (F)
	TCCTAATATAACTCCAGTAGTACCTCC	Fragment 1 (R)
	CCTATGCATTTCTTAGGGTTTTCC	Fragment 2 (R)
	AGGAATTGATATTAGACGTCAGAAAGTAC	Fragment 3 (R)
	CAAATGAGTTATTGGGGAGC	Fragment 3 (R)
	GGAATAGGAAAGATTAACCGCTATC	Fragment 3 (R)
***cox3* PCR**[c]	ACAGGATTAGATACCCTGG	cox3 (F)
	ACTGTCAGCTAAAACGTATC	cox3 (R)

[a]Due to its unique mitochondrial genome structure as well as the inability to successfully perform TIR PCR, *B. conradae* required alternative PCR assays to obtain additional mitochondrial genome sequence

[b]Additional primers were designed to obtain complete bi-directional sequencing of mitochondrial fragments

[c]Primers designed to amplify *cox3* from *B. conradae* yielded a *cox3*-like amplicon

[d](F)=Forward Primer, (R)=Reverse Primer

[e]PCR amplicons required cloning for full sequence resolution
