# Supplementary material for: Mitochondrial Genome Sequences and Structures Aid in the Resolution of Piroplasmida phylogeny
Source: PLoS One. 2016 Nov 10;11(11):e0165702. doi: 10.1371/journal.pone.0165702 (PMC5104439; doi:10.1371/journal.pone.0165702)
Supplement: S7 Table — (PDF) [file pone.0165702.s016.pdf]

S7 Table. Primers utilized in additional *B. microti*-like sp. PCR assays.

Purpose	Sequence	Amplicon[c]
**Mitochondrial genome PCR amplification[a]**	TATTAGCTACCTTTGGCTATC	Fragment 1 (F)
	AGTATTAGCACATACACTTCTGG	Fragment 1 (R)
	GGAAGTGGWACWGGWTGGAC	Fragment 2/cox1 (F)
	TTCGGTATTGCATGCCTTG	Fragment 2/cox1 (R)
	ACAAAGACTAATGCTTGAAGTGG	Fragment 3 (F)
	GTAACTCAGTCAGACCTTTG	Fragment 3 (R)
	TTAGTGAAGGAACTTGACAGGT	Fragment 4/cytb (F)
	CGGTTAATCTTTCCTATTCCTTACG	Fragment4.cytb (R)
	CTCGATATTAATCTTAAAGTACAGGAC	Fragment 5 (F)
	ATCTAGTGCCAGCAGTAG	Fragment 5 (R)
	AGCTTACTAATGAATTGTCTCG	Fragment 6 (F)
	CCTTCTATAATTATGCTAATTTCAGTAG	Fragment 6 (R)
**Additional sequencing[b]**	AGTATTAGCACATACACTTCTGG	Fragment 2/cox1 (R)
	CATCTGATATCTACATGATGGC	Fragment 4/cytb (F)
	CATTTCGTAGAAGCTGTATGG	Fragment 5 (F)
	TTCAGCTACAAGTTCACTGTC	Fragment 5 (F)
	TTGCTCACACAGTCAATACG	Fragment 5 (R)
	ACTCATATCTATTACCACTATAGGC	Fragment 5 (R)

[a]Due to its mitochondrial genome structure, *B. microti*-like sp. required alternative PCR assays to obtain additional mitochondrial genome sequence

[b]Additional primers were designed to obtain complete bi-directional sequencing of mitochondrial fragments

[c](F)=Forward Primer, (R)=Reverse Primer
