# Supplementary material for: Mitochondrial Genome Sequences and Structures Aid in the Resolution of Piroplasmida phylogeny
Source: PLoS One. 2016 Nov 10;11(11):e0165702. doi: 10.1371/journal.pone.0165702 (PMC5104439; doi:10.1371/journal.pone.0165702)
Supplement: S8 Table — Gene/fragment coordinates with a white background are coded on the sense strand, while those highlighted in yellow are on the antisense strand. Some genes/fragments were not identified in some mitochondrial genomes (indicated in green and with “No ID”) while others were duplicated for some mitochondrial genomes (indicated in blue). Coordinates initially reported in separate studies are not reported in this table (noted in gray, “previously published”). Genes/fragments that were identified for all species were included in phylogenetic analysis (highlighted in red). (PDF) [file pone.0165702.s017.pdf]

S8 Table. Locations of protein-coding genes and rRNA fragments within *Piroplasmida* mitochondrial genomes. Gene/fragment coordinates with a white background are coded on the sense strand, while those highlighted in yellow are on the antisense strand. Some genes/fragments were not identified in some mitochondrial genomes (indicated in green and with "No ID") while others were duplicated for some mitochondrial genomes (indicated in blue). Coordinates initially reported in separate studies are not reported in this table (noted in gray, "previously published"). Genes/fragments that were identified for all species were included in phylogenetic analysis (highlighted in red).

Species and GenBank Accession Number

Gene	*Cytauxzoon felis* KC207821	*Babesia canis* KC207822	*Babesia rossi* KC207823	*Babesia vogeli* KC207825	*Babesia sp.* Coco KC207824	*Babesia conradae* KC207826	*Babesia microti-like sp.* KC207827	*Babesia gibsoni* AB499087	*Babesia bigemina* AB499085	*Babesia caballi* AB499086	*Babesia bovis* AB499088	*Theileria parva* AB499089	*Theileria annulata* NW001091933	*Theileria orientalis* AB499090	*Theileria equi* AB499091	*Babesia rodhaini* AB624357	*Babesia microti (linear)** AB624353	*Babesia microti (circular)* FO082868
cox1	25-1455	19-1452	44-1477	1-1317	1-1317	1-1317	1-1382											
cox3	2899-2258	2915-2274	2943-2299	2781-2140	2793-2152	No ID^	3321-3986											
cytb	5447-4356	5476-4385	5494-4403	5343-4250	5351-4260	5346-4249	1989-3107											
LSU1	3052-3350	3067-3365	3094-3391	2933-3231	2947-3247	2925-3205	4497-4775											
LSU2	4353-4319	4379-4345	4397-4363	4244-4210	4256-4222	4229-4196	3108-3139						**Previously published**					
LSU3	3472-3362	3486-3376	3512-3402	3352-3242	3368-3258	3327-3216	4891-4782											
LSU4	5620-5701	5661-5742	5664-5754	5526-5605	5533-5612	5529-5608	1854-1774											
LSU5	5550-5485	5585-5514	5600-5532	5450-5381	5459-5389	5455-5384	1982-1916											
LSU6	4293-4251	4320-4278	4338-4296	4185-4143	4197-4155	4171-4129	3169-3189											
LSUA	4025-3858	4050-3881	4069-3900	3915-3746	3928-2759	3888-3727	5477-5637	4092-3923	4099-3937	4094-3925	4161-3993	4107-3939	4101-3934	1849-2012	3054-2893	6492-6657	9699-9863*	10823-10983
LSUB	3623-3646	3636-3659	3662-3685	3502-3525	3518-3541	3480-3503	5069-5046	3690-3713	3936-3719	3688-3711	3767-3790	3700-3724	3694-3717	2257-2234	2712-2735	6083-6063	9293-9270*	10420-10397
LSUC	1789-1804	No ID	No ID	No ID	1673-1688	1868-1853	No ID	1848-1863	1853-1868	1846-1861	No ID	1858-1873	1850-1865	No ID	No ID	No ID	No ID	No ID
SSUA	3622-3519	3635-3532	3661-3558	3501-3398	3517-3412	3479-3378	5042-4951	3684-3586	3695-3590	3687-3582	3766-3663	3699-3594	3693-3588	2258-2363	2665-2588	6048-5956	9266-9175*	10393-10302
SSUB	4135-4027	4162-4054	4181-4074	4027-3919	4040-3932	4007-3900	5354-5419	4204-4096	4218-4110	4207-4099	4275-4167	4216-4108	4211-4103	1734-1842	3169-3061	6379-6444	9580-9645*	10700-10765
SSUD	3755-3689	3778-3712	3797-3731	3643-3577	3656-3590	3618-3556	5192-5130	3820-3754	3834-3768	3822-3756	3890-3824	3833-3766	3827-3761	2121-2187	1705-1638	6197-6130	9419-9353*	10539-10474
SSUE	2062-2024	2067-2030	2091-2054	1931-1894	1941-1903	1708-1672	4200-4239	2116-2078	2122-2084	2113-2075	2193-2156	2127-2089	2120-2082	3829-2865	3617-3581	5289-5250	8307-8346*	9543-9582
SSUF	1805-1865	1817-1877	1843-1903	1684-1744	1689-1749	1852-1792	4463-4408	1864-1924	1869-1929	1862-1922	1951-2011	1874-1934	1866-1926	4082-4022	2028-2084	5440-5385	8564-8509*	9799-9744
RNA1	3779-3857	3802-3880	3821-3899	3667-3745	3680-3758	3646-3722	5693-5662	3844-3922	3858-3936	3846-3924	3929-3992	3860-3938	3855-3933	2091-2013	2828-2892	6744-6678	9948-9888*	11008-11068
RNA2	3057-2991	3072-3006	3099-3033	2938-2872	2952-2886	2911-2845	No ID	3119-3053	3133-3069	3122-3056	3200-3134	3131-3065	3123-3057	2834-2900	3441-3375	4123-4170	7282-7328*	8517-8562
RNA6	1608-1549	1612-1553	1638-1579	1479-1420	1481-1422	2054-2112	1554-1496	1661-1602	1657-1598	1651-1592	1744-1685	1677-1618	1669-1610	4279-4338	1922-1975	1825-1765	2452-2391*	4155-4094
RNA7	1647-1723	1648-1727	1674-1753	1515-1594	1517-1596	2008-1936	1682-1611	1697-1776	1693-1772	1687-1766	1780-1859	1713-1792	1705-1784	4239-4162	1887-1808	1955-1882	2565-2512	4285-4214
RNA8	2990-2906	3005-2913	3032-2943	2871-2779	2885-2797	No ID	No ID	3052-2960	3064-2976	3055-2967	3133-3050	3064-2973	3056-3965	2901-2992	3374-3290	No ID	No ID	No ID
RNA9	4165-4136	4190-4163	4209-4182	4055-4028	4068-4041	No ID	No ID	4232-4205	4246-4219	4235-4208	4303-4276	4245-4217	4240-4212	1704-1733	3198-3170	No ID	No ID	No ID
RNA11	2256-2205	2265-2208	2282-2233	2120-2072	2136-2088	No ID	No ID	2304-2255	2317-2266	2306-2257	2389-2338	2332-2272	2316-2264	3634-3685	No ID	No ID	No ID	No ID
RNA14	2006-1963	2006-1971	2030-1994	1870-1836	1879-1845	1647-1614	No ID	2060-2047	2060-2026	2051-2017	2132-2100	2071-2029	2064-2022	3883-3922	No ID	No ID	No ID	No ID
RNA15	1724-1754	1730-1757	1755-1782	1597-1624	1603-1630	1933-1906	1703-1729	1779-1809	1779-1806	1769-1796	1862-1889	1793-1823	1785-1815	4161-4131	1534-1560, 6713-6687	2012-2040	2606-2634	4305-4337
RNA17	2156-2118	2161-2123	2185-2147	2025-1987	2039-2001	1419-1450, 2288-2257	4104-4141	2208-2170	2218-2180	2209-2171	2290-2252	2224-2186	2216-2178	3734-3772	3762-3724	5088-5124	8212-8249*	9447-9484
RNA18	1484-1460	1480-1456	1506-1482	1346-1322	1346-1322	1518-1494, 2189-2212	1415-1391	1534-1510	1523-1499	1519-1495	1620-1596	1552-1528	1545-1521	4407-4431	1732-1708	1673-1649	2309-2285*	4011-3987

^Although a *cox3*-like gene was amplified, its location relative to the mitochondrial genome is unknown

*Provided by Kenji Hikosaka
