# Supplementary material for: Mitochondrial Genome Sequences and Structures Aid in the Resolution of Piroplasmida phylogeny
Source: PLoS One. 2016 Nov 10;11(11):e0165702. doi: 10.1371/journal.pone.0165702 (PMC5104439; doi:10.1371/journal.pone.0165702)
Supplement: S2 Fig — Because attempts at TIR amplifications were unsuccessful, primers were designed to amplify near full length mitochondrial genomes of species in five overlapping fragments. Primers for fragments 0–4 are indicated with arrows (forward primers: F0-F4, reverse primers: R0-R4). Protein-coding genes (cox1, cox3, and cytb) are indicated in white. Large subunit rRNA fragments are in light gray, small subunit rRNA fragments are in dark gray, and miscellaneous conserved RNA fragments are in black. (PDF) [file pone.0165702.s002.pdf]

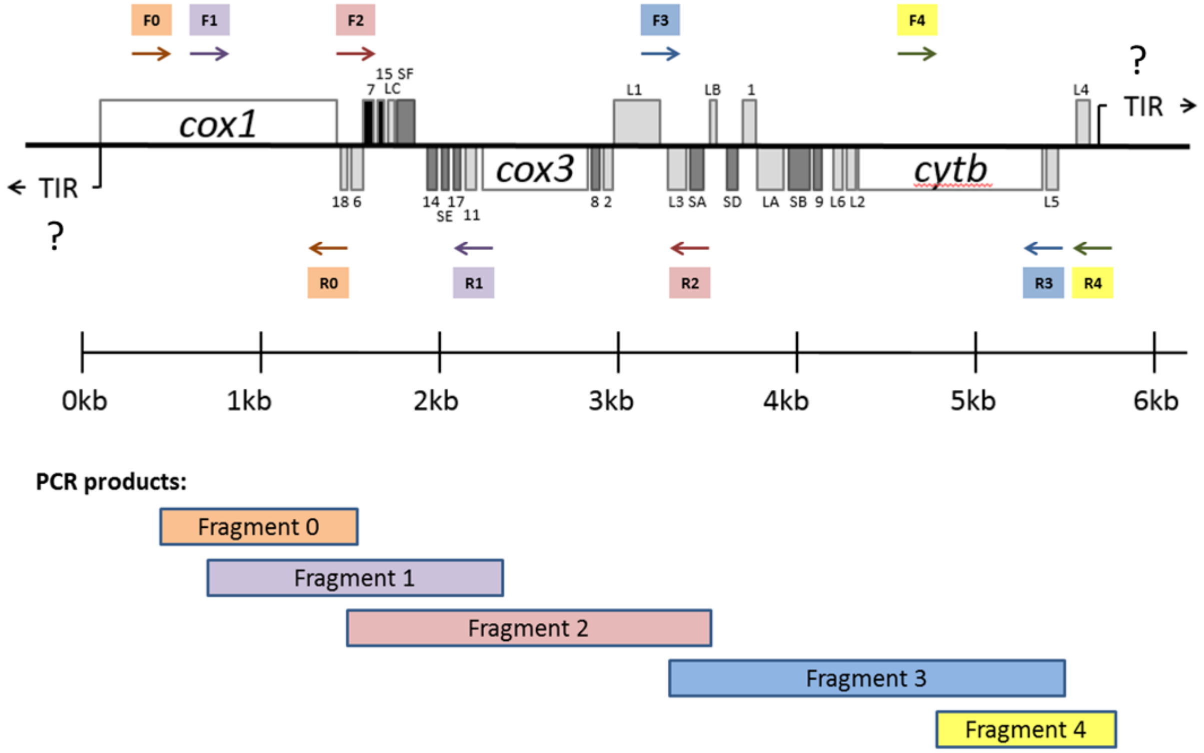

S2 Figure. Schematic of PCR amplification of *B. vogeli* and *Babesia sp.* Coco mitochondrial genomes. Because attempts at TIR amplifications were unsuccessful, primers were designed to amplify near full length mitochondrial genomes of species in five overlapping fragments. Primers for fragments 0-4 are indicated with arrows (forward primers: F0-F4, reverse primers: R0-R4). Protein-coding genes (*cox1*, *cox3*, and *cytb*) are indicated in white. Large subunit rRNA fragments are in light gray, small subunit rRNA fragments are in dark gray, and miscellaneous conserved RNA fragments are in black.
